# Lateral opening in the intact β-barrel assembly machinery captured by cryo-EM

**DOI:** 10.1038/ncomms12865

**Published:** 2016-09-30

**Authors:** Matthew G. Iadanza, Anna J. Higgins, Bob Schiffrin, Antonio N. Calabrese, David J. Brockwell, Alison E. Ashcroft, Sheena E. Radford, Neil A. Ranson

**Affiliations:** 1 Astbury Centre for Structural Molecular Biology, School of Molecular and Cellular Biology, University of Leeds, Mount Preston Street, Leeds LS2 9JT, UK

## Abstract

The β-barrel assembly machinery (BAM) is a ∼203 kDa complex of five proteins (BamA–E), which is essential for viability in *E. coli*. BAM promotes the folding and insertion of β-barrel proteins into the outer membrane via a poorly understood mechanism. Several current models suggest that BAM functions through a ‘lateral gating' motion of the β-barrel of BamA. Here we present a cryo-EM structure of the BamABCDE complex, at 4.9 Å resolution. The structure is in a laterally open conformation showing that gating is independent of BamB binding. We describe conformational changes throughout the complex and interactions between BamA, B, D and E, and the detergent micelle that suggest communication between BAM and the lipid bilayer. Finally, using an enhanced reconstitution protocol and functional assays, we show that for the outer membrane protein OmpT, efficient folding *in vitro* requires lateral gating in BAM.

## Abstract (web summary)

The β-barrel assembly machinery (BAM complex) is a key mediator of outer membrane protein biogenesis in Gram-negative bacteria. Here the authors report a cryo-EM structure of the intact BAM complex that suggests that lateral gate opening is a necessary part of the BAM functional cycle.

## Graphical abstract

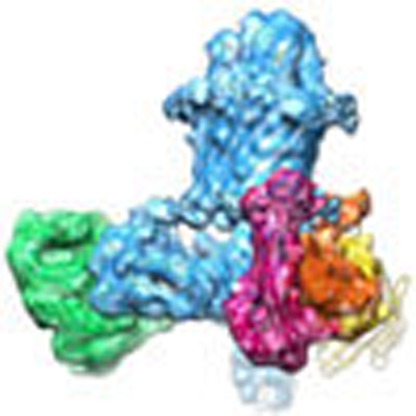

β-Barrel outer membrane proteins (OMPs) perform diverse functions in the outer membrane of Gram-negative bacteria and are critical for viability and pathogenesis^1^,^2^. Folding and insertion of OMPs into the outer membrane is mediated in *Escherichia coli* by the β-barrel assembly machinery (BAM) complex, via a mechanism that remains unresolved^3^,^4^,^5^. An understanding of how BAM-mediated OMP folding and insertion occurs will provide insight into OMP biogenesis in *E. coli* and potentially of homologous proteins in the outer membranes of mitochondria^6^ and chloroplasts^7^. In addition, as BAM is surface located, essential and conserved, it is an attractive potential target for the development of novel antibacterials^8^. Recent evidence demonstrated that deletion of BamB from *Klebsiella pneumonia* attenuated virulence *in vivo*^9^, highlighting the potential of BamB as an antibiotic target, which may be less prone to selection pressure than essential proteins^10^.

The *E. coli* BAM complex has a molecular mass of ∼203 kDa and consists of five proteins (BamA–E). Its core component is the Omp85-family member BamA, which contains a carboxy-terminal, membrane-embedded, 16-stranded β-barrel domain and five polypeptide transport-associated (POTRA) domains at its amino terminus, which project into the periplasm.

The other four subunits (BamB–E) are accessory lipoproteins^11^ ranging from 12 to 41 kDa in mass and attached to the membrane by N-terminal lipid anchors. *In vivo*, only BamA and BamD are essential, whereas the others are thought to modulate the substrate specificity and activity of the complex^12^. Deletion of BamB, BamC or BamE results in a variety of outer membrane defects. Consistent with this, all five BAM subunits are required for maximal OMP folding activity *in vitro*^13^,^14^,^15^.

*In vivo*, OMPs are synthesized on cytoplasmic ribosomes, translocated into the periplasm via the Sec translocon and transported to the outer membrane by the periplasmic chaperones Skp and SurA^16^,^17^. Thereafter, they are delivered to the BAM complex for insertion, folding and assembly into the outer membrane. The molecular mechanisms underpinning these processes remain unclear, but OMP insertion is independent of ATP, proton gradients or other apparent driving forces^18^. Many different models have been suggested for the mechanism of action of BAM. The complex may function as a ‘disruptase', destabilizing the membrane to assist in OMP insertion^19^,^20^. BAM may also take a more active role, making interactions with the substrate^4^, including the possible formation of a hybrid barrel in which the substrate protein donates β-strand(s) to expand the BamA barrel^20^. Oligomerization of BamA alone occurs *in vitro*^21^, although recent evidence indicates that a single copy of the whole BAM complex is sufficient for the assembly of the autotransporter EspP^14^. However, spatial clustering of BAM complexes may be functionally relevant, as BAM has been observed in ∼0.5 μm ‘OMP islands' *in vivo*^22^. The molecular mechanisms underlying BAM activity may also vary depending on the substrate^12^ or membrane environment^23^, and a single mechanism may be insufficient to explain all aspects of BAM activity.

Crystal structures of individual BAM subunits^24^,^25^,^26^,^27^, sub-complexes^28^,^29^,^30^,^31^,^32^ and the complete complex^33^,^34^ have recently been reported. These show that BAM can occupy multiple conformations, which differ in the structure of the BamA β-barrel, the arrangement of the BamA POTRA domains relative to the β-barrel, the positions of BamB–E relative to BamA and the implied position of the membrane. The role these conformational states play in OMP folding and which, if any, represent ‘resting' or ‘active' states of the complex for OMP binding and membrane insertion remains unclear.

Recent crystallographic structures demonstrate that the β-barrel of BamA can exist in two distinct conformations. In the first, the β-barrel is sealed on its extracellular face and open to the periplasm (and hence is presumably an acceptor state for OMPs). This conformation has been termed the ‘inward open' state^33^. A second conformation has recently been observed in the BAM complex^33^,^34^ in which the BamA β-barrel is distorted by the displacement of three loops on its extracellular face, leading to the separation of the β1 and β16 strands. This opens the barrel to the outer leaflet of the outer membrane and the extracellular space, a so-called ‘lateral open' state^32^,^33^. For clarity we will henceforth describe the first of these conformations as a ‘lateral closed' rather than ‘inward open' state, allowing the description of β-barrel dynamics as a simple opening and closing. Thus far, ‘lateral open' conformations have only been observed in BAM structures, which lack the 42 kDa, β-propeller protein BamB^32^,^33^, raising the possibility that changes in BamA may represent a gating reaction driven by BamB binding and dissociation. The crystal structure of the BamA homologue TamA, which is involved in autotransporter assembly, also exhibits incomplete hydrogen bonding and an opening between strands β1 and β16, suggestive of lateral gating^35^. The TAM complex lacks a BamB component, but TamA is thought to interact with the inner membrane-anchored TamB^36^ whose role in gating, if any, is unknown.

To investigate the conformational plasticity of BAM in more detail, we have analysed its structure using cryo-electron microscopy (cryo-EM) and complemented these data with functional assays of OMP folding by the same complex *in vitro*. Here we present the cryo-EM structure of the intact BAM complex solubilized in *n*-dodecyl-β-D-maltopyranoside (DDM) at 4.9 Å resolution, which captures the intact complex in a ‘lateral open' conformation for the first time. This solution structure of BAM shows conformational rearrangements throughout the complex relative to previous crystallographic structures in both the presence and absence of BamB^32^,^33^,^34^. The structure also describes interactions between four of the five BAM subunits with the detergent micelle, suggesting how they might interact with the lipid bilayer. The same BAM preparation dialysed into proteoliposomes shows unprecedented levels of OmpT folding activity. We have also performed functional assays for a BAM complex in which BamA has been mutated to contain a single pair of cysteine residues that are predicted to form a disulfide bridge across the BamA ‘gate'. Such a disulfide should prevent the complex adopting a ‘lateral open' conformation. Indeed, we show that oxidation causes a decrease in BAM activity, which is reversed in reducing conditions, confirming previous suppositions based on *in vivo* data^33^,^37^ that gating of the BamA barrel is required, at least in part, for BAM function *in vitro*.

## Results

### Characterization of the purified BamABCDE complex

**Figure 1:**
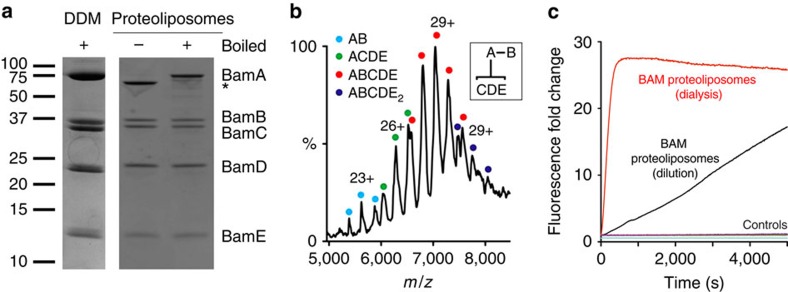
Characterization of the intact BAM complex and optimization of its activity in *E. coli* polar lipids. (**a**) SDS–polyacrylamide gel showing that all five subunits are present in the purified BAM complex reconstituted into DDM micelles (analysed after boiling (+)) or into liposomes formed from *E. coli* polar lipids by extensive dialysis. The unboiled and boiled samples show a differential electrophoretic mobility (band-shift) for BamA, consistent with this subunit being folded in the proteoliposomes. The full gels are shown in Supplementary Fig. 10. (**b**) Intact electrospray ionization–mass spectrum of the BAM complex. Charge states from the intact complex, as well as subcomplexes formed by gas phase ionization (inset) are indicated. The intact complex elutes as a single peak on size exclusion chromatography, indicating that it is intact in solution (Supplementary Fig. 1). (**c**) Optimization of BAM complex-containing proteoliposomes for activity. Denatured OmpT in solution in the presence of a seven-fold molar excess of SurA was added to liposomes that are empty or contain BAM. Successful folding results in an increase in fluorescence by cleavage of the fluorogenic substrate. Controls were performed in the absence of OmpT (green), SurA (blue), fluorogenic peptide (cyan) and using empty liposomes (pink). BAM proteoliposomes generated by dilution or dialysis are compared (see Methods).

The full BAM complex, expressed as the product of five genes encoded on a single plasmid, was purified as previously reported^14^ (Fig. 1 and Supplementary Fig. 1a–c). The presence of all five subunits in an ∼1:1:1:1:1 molar ratio was confirmed by SDS–polyacrylamide gel electrophoresis (SDS–PAGE; Fig. 1a), gel filtration (Supplementary Fig. 1a,d,e) and non-covalent ‘native' mass spectrometry (MS; Supplementary Table 1 and Fig. 1b). The resulting spectra revealed that the complex remains largely intact after introduction into the gas phase, with a charge-state distribution corresponding to BamABCDE being the predominant species in the mass spectrum (Fig. 1b; observed mass 203,456±22 Da, predicted mass 203,218 Da; Supplementary Table 1). A minor contribution to the spectrum is a distribution corresponding to the intact complex with an additional E subunit bound (Fig. 1b). Previous analyses of the BAM complex made by reconstitution of purified BamAB and BamCDE subcomplexes^13^ could not resolve whether one or two copies of BamE were present in the complex and BamE dimerization has also been observed *in vitro*^23^,^24^,^38^. Several sub-complexes are also observed, notably BamAB and BamACDE (Fig. 1b and inset, Supplementary Table 1), likely to be a result of low levels of dissociation of the complex in the gas phase, consistent with the known modular architecture of the BAM complex^13^,^39^. The molecular masses of the individual BAM subunits were also confirmed by denaturing MS (Supplementary Fig. 2a,b), which also allowed the carbon-chain lengths of the N-terminal lipid anchors for BamB, C, D and E to be determined unambiguously (Supplementary Fig. 2c,d).

The purified BAM complex was reconstituted into liposomes formed from *E. coli* polar lipids by dialysis. We used a previously described assay for BAM activity in OMP folding, in which a self-quenching fluorogenic reporter peptide is cleaved by the correctly folded/membrane inserted endoprotease OmpT (see Methods)^13^,^40^. The extent of OmpT folding was monitored by measuring the fluorescence increase over time (as a result of peptide cleavage; Fig. 1c). Previous work has shown that pre-incubation of OmpT with SurA is required for efficient BAM-mediated folding, presumably to maintain OmpT in a soluble, folding-competent state^13^,^15^,^41^. Consistent with this, removal of SurA (or OmpT or BAM from the assay) eliminates the fluorescence increase associated with BAM-mediated folding of OmpT (Fig. 1c and Supplementary Fig. 3).

Previous protocols for *in vitro* reconstitution of BAM into liposomes used dilution of detergent-solubilized BAM in the presence of *E. coli* polar lipid extract to create proteo-liposomes^13^,^14^. Here we report an alternative method using dialysis to generate BAM-containing proteoliposomes, which have dramatically greater activity than those generated by dilution (Fig. 1c), likely to be due to a greater efficiency of reconstitution. Together, these results demonstrate the preparation of proteoliposomes containing an intact and highly functional BAM complex.

### BamABCDE is in a ‘lateral open' conformation

**Figure 2:**
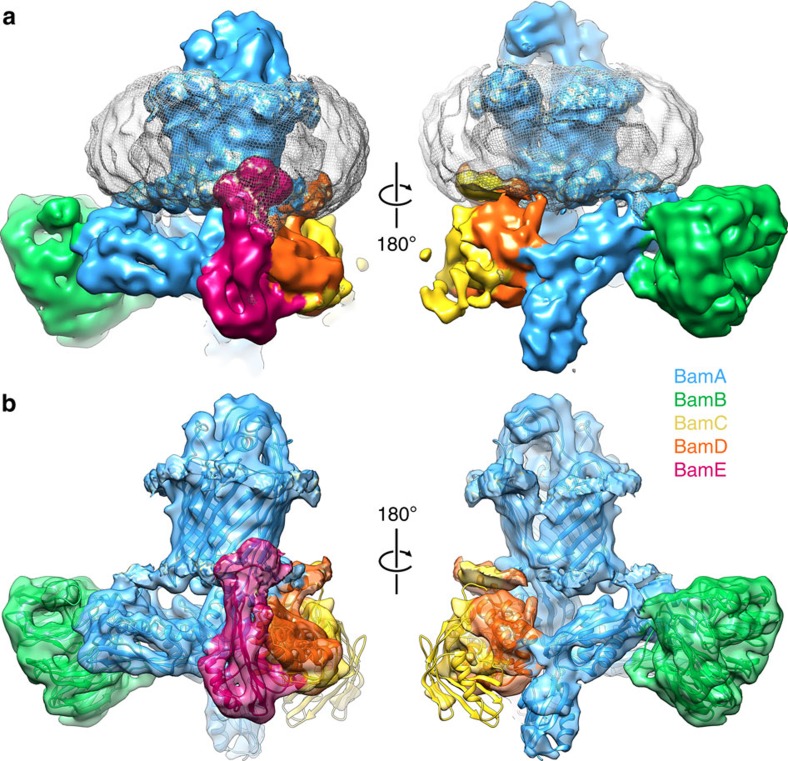
Cryo-EM structure of the BAM complex. (**a**) Views of the front and back face of the cryo-EM structure of the intact BAM complex at 4.9 Å resolution. BamA is coloured blue, BamB in green, BamC in yellow, BamD in orange and BamE in magenta. Density corresponding to the micelle of *n*-dodecyl-β-D-maltopyranoside (DDM) in the structure is shown as a pale grey mesh. (**b**) Flexible fitting of a hybrid X-ray structure into the EM density. The views and colouring are identical, but density for the micelle has been masked and the EM density made transparent, showing the fitted pseudo-atomic model within. This colour scheme is maintained for all further figures showing the cryo-EM structure. The figure was made using UCSF Chimera^64^.

Using cryo-EM and single-particle image processing, we next determined a structure for the intact BamABCDE complex solubilized in DDM, at 4.9 Å resolution (Supplementary Fig. 4). The cryo-EM map contains density for an unambiguous single copy each of BamA, B, D and E, as well as the N-terminal ‘lasso' of BamC. The density for the N-terminal globular domain of BamC is somewhat weaker, consistent with previous observations of disorder in this part of the complex^32^. The map also contains a large, uniform, relatively diffuse, doughnut-shaped density consistent in both size and appearance with a detergent micelle^42^, which, as expected, surrounds the BamA β-barrel (Fig. 2a).

To explore the conformation and compositional heterogeneity of the data set, we performed extensive three-dimensional classification^43^ but did not identify any subsets of particles representing either different conformations or different BAM sub-complexes. Specifically, there is no evidence of a complex that lacks BamB^32^. Similarly, no evidence could be found for a complex in which the BamA β-barrel was in a ‘laterally closed' conformation barrel. Although complexes with variable lengths of BamC have been observed previously^33^, and to search for such conformational variability, the particles were masked and a focused classification performed solely on the N-terminal globular domain of BamC. This again failed to detect any alternate conformations, suggesting the particles originate from a homogenous pool.

**Figure 3:**
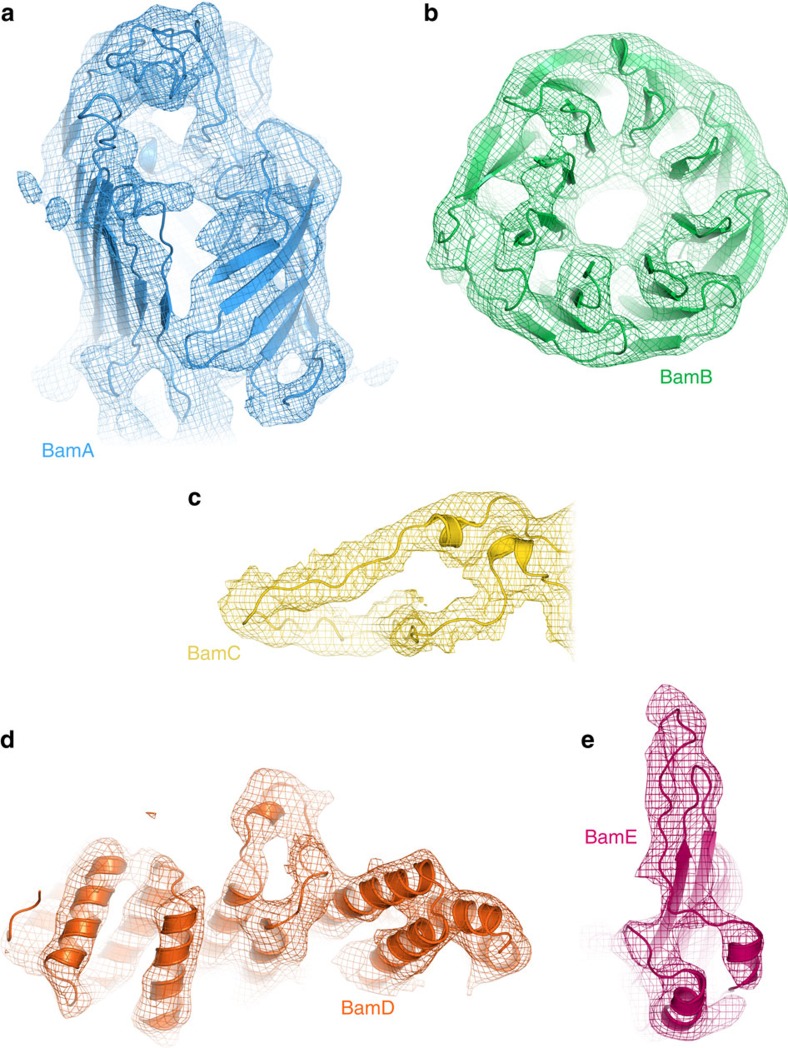
Resolution of secondary structure in individual subunits of the BAM complex. The EM map has a mean resolution of 4.9 Å, allowing resolution of secondary structure throughout the BAM structure, including (**a**) the β-barrel of BamA, which is in a ‘lateral open' conformation, (**b**) the β-propeller of BamB, in which β-sheets are well resolved, (**c**) the ‘lasso' of BamC, (**d**) the α-helical region of BamD and (**e**) α-helices and β-sheets in BamE. All panels were made using PyMOL v1.7 (ref. 65).

A preliminary examination of the density, both visually and by rigid body fitting of existing crystal structures, both with and without BamB, (5EKQ^32^, 5D0O^33^, 5D0Q^33^ and 5AYW^34^) confirmed that the gross architecture of the complex and the structures of the individual components is similar to previous crystallographic structures. However, no single X-ray structure was able to fit the EM density satisfactorily, especially in the region containing BamAB. Most notably, the β-barrel of BamA is in a ‘lateral open' conformation despite the presence of BamB (Figs 2 and 3a, and Supplementary Table 2) in marked contrast with previous crystallographic data, which suggested an incompatibility of BamB binding with a lateral open conformation^32^,^33^. To enhance the interpretability of the EM density, we therefore constructed a hybrid atomic model consisting of the ‘lateral open' β-barrel of BamA from 5D0Q^33^ (which lacks BamB) and the POTRA region of BamA from a ‘lateral closed' structure, which does contain BamB (5D0O^33^). The structure for each of the accessory proteins BamB–E was taken from a variety of published X-ray structures. A pseudo-atomic model was then generated with flexible fitting by molecular dynamics^44^ and Monte Carlo simulations^45^ into the 4.9 Å resolution density (Figs 2b and 3). The striking difference in the cryo-EM structure, compared with all previous crystallographic structures, is that in the intact BAM complex, the BamA β-barrel is unambiguously in the ‘lateral open' conformation^32^,^33^, the first time this conformation has been observed in the presence of BamB.

### Conformational changes in the BamA POTRA domains

**Figure 4:**
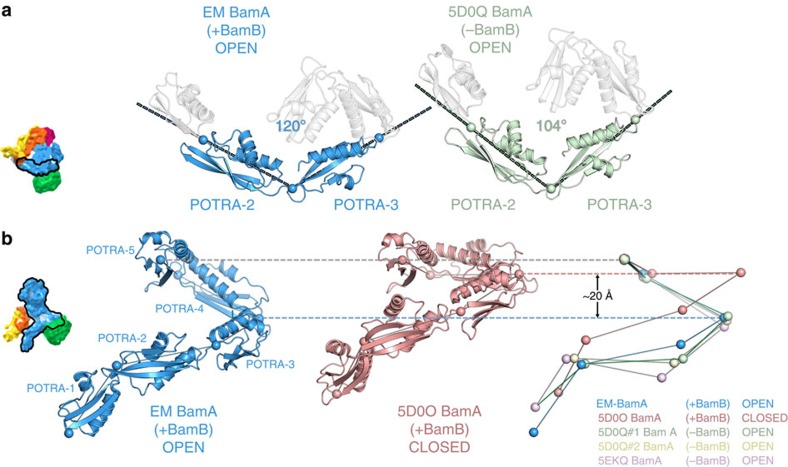
The effect of BamB binding and β-barrel conformation on the BamA POTRA domains. (**a**) The presence of BamB correlates with a more obtuse angle between POTRA domains 2 and 3. On the left, is the EM structure (‘lateral open', +BamB; blue), and on the right 5D0Q^33^ (a ‘lateral open', -BamB, X-ray structure; pale green). The view is from outside the bacterial cell, looking approximately down the axis of the β-barrel (see thumbnail image). Both structures have the BamA β-barrel in a ‘lateral open' conformation; thus, the barrel opening and a wide POTRA 2–3 angle do not correlate, but BamB binding and a wide POTRA angle do correlate. The remaining POTRA domains are shown in pale grey. The angle is measured between identical points in the hinge regions between each POTRA domain, indicated by spheres. (**b**) Vertical extension of the POTRA domains correlates with β-barrel state. The open barrel of the EM structure (‘lateral open', +BamB; blue) and all ‘lateral open' X-ray structures, correlates with a vertically extended conformation of the POTRA chain, whereas the ‘lateral closed' barrel of 5D0O (+BamB; pink) has a much more compact POTRA chain. Both structures contain BamB; hence, BamB binding does not appear to correlate with the extension of the POTRA chain. Thumbnail images are in the appropriate view and coloured with the same colour scheme as Fig. 2. For overall comparisons of BamA, see Supplementary Fig. 9.

Although the conformation of the BamA β-barrel in the cryo-EM structure obtained here is similar to previous ‘lateral open' structures obtained by crystallography^32^,^33^, the five POTRA domains have undergone a large conformational rearrangement relative to the lateral open structure^33^,^34^ (Fig. 4). Indeed, the N-terminal POTRA domain (POTRA 1) appears to be the most mobile as evidenced by its weaker density compared with POTRA domains 2–5 (Supplementary Fig. 5). Although POTRA 1 is most displaced from its position in previous BamA structures, analysis of the relative positions and angles between the POTRA domains, in the EM structure and all crystallographic structures to date (5EKQ^32^, 5D0O^33^, 5D0Q^33^ and 5AYW^34^), shows that this displacement occurs as the result of a complex rearrangement of the POTRA domains further up the chain (Fig. 4). Looking approximately down the axis of the BamA barrel (from the extracellular face), the angle between POTRA domains 2 and 3 is variable, consistent with previous suggestions that this region is capable of hinge-like motions^46^. This angle is wide (∼120°) in structures with BamB present and a ‘lateral closed' BamA β-barrel conformation, and more acute (∼104° to ∼110°) in structures in a ‘lateral open' conformation. Previously, all such ‘lateral open' structures have lacked BamB, but the EM structure does not. The conformation of this part of the POTRA chain in the EM structure is thus more similar to the ‘lateral closed' conformation of 5D0O^33^. The simultaneous presence of BamB and a wide POTRA 2–3 angle in the ‘lateral open' EM structure demonstrates that conformational gating of the BamA barrel is not dependent on the presence or absence of BamB. An analysis of the tight crystal packing in previous ‘lateral open' structures suggests that the lack of BamB in all such crystallographic studies may result from degradation of BamB and preferential crystallization of the ‘BamB-less' form, which may be more easily accommodated in the crystal lattice (Supplementary Fig. 6).

As well as changes in the arrangement of the POTRA domains looking along the axis of the BamA β-barrel, large rearrangements also occur parallel to that axis. These result in an extension of the POTRA domains by up to ∼20 Å perpendicular to the putative position of the membrane (Fig. 4b). This extension is primarily determined by the angle of POTRA domains 4 and 5 relative to the plane of the membrane. In the EM structure, this angle is similar to other ‘lateral open' structures, all of which lack BamB (see Supplementary Table 2). All of these structures differ from the ‘lateral closed' structure. Therefore, POTRA extension does not appear to be affected by the presence or absence of BamB. The conformation of BamA in the EM structure is thus a hybrid between previous ‘lateral open' and ‘lateral closed' states. Together, with previous crystallographic structures, this hints at the dynamic nature of BamA, which presumably reflects a role for large-scale conformational movements in BAM function.

### Interactions between BAM subunits and the detergent micelle

A further difference between the cryo-EM structure presented here and all previous crystallographic structures is the presence of the detergent micelle acting as an analogue of the lipid bilayer in which BAM resides. Interestingly, this micelle surrounds the β-barrel of BamA (Fig. 2a), encapsulating all of the hydrophobic residues that anchor the β-barrel in the lipid bilayer (see Supplementary Fig. 7).

**Figure 5:**
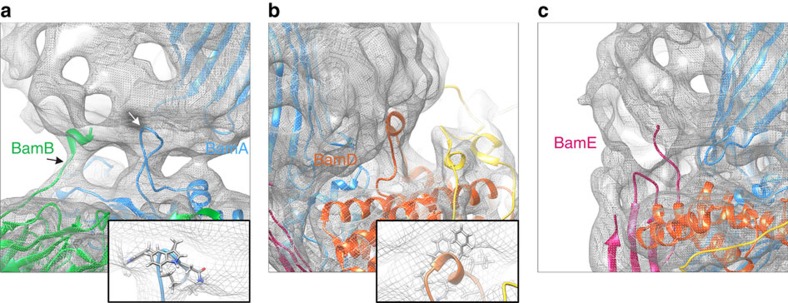
Interactions between BAM components and the detergent micelle. (**a**) A hydrophobic loop within the body of BamA POTRA 3 (blue, white arrow) is buried within the micelle (grey mesh), with details of the hydrophobic residues inset. The N terminus of BamB (green, black arrow), which is unmodelled in the X-ray structures of BAM and its subcomplexes^32^,^33^,^34^, also dips into the micelle. (**b**) A hydrophobic 3_10_ helix in BamD (orange) inserts into the micelle, with hydrophobic residues buried in the hydrocarbon tail groups of the detergent (see inset) and polar residues flanking the helix placed to interact with the polar head groups of the detergent. (**c**) The N terminus of BamE (magenta), which is the site of the lipid anchor, also inserts into the micelle, well away from the body of the BamA β-barrel.

Additional contacts are observed between regions of BamA outside the β-barrel and the micelle, along with additional bridges of density from BamB, BamD and BamE. For BamA, this contact is formed by a loop (residues 196–214 in POTRA 3; white arrow in Fig. 5a), which contains a hydrophobic sequence flanked by charged residues (RDE-**VPWWNVVG**-DRK) and is buried in the detergent micelle. For BamB, the contact is made at the N terminus of the protein (black arrow in Fig. 5a), which is unmodelled in the crystal structures of BAM complexes but not in isolated BamB^32^,^33^,^34^, and which contains either 3 × C_16_, or 2 C_16_ and 1 C_18_, lipid anchors (Supplementary Fig. 2). Further interactions between segments of BamD (Fig. 5b) and the N terminus of BamE (Fig. 5c), and the micelle are also observed. Thus, we observe a number of distinct interactions between hydrophobic segments of polypeptide chain, or lipid anchor, and the detergent micelle, suggesting an intimate and extensive interaction between protein and membrane that extends far beyond the obvious interactions made by the BamA β-barrel.

### The conformation of BamCDE

**Figure 6:**
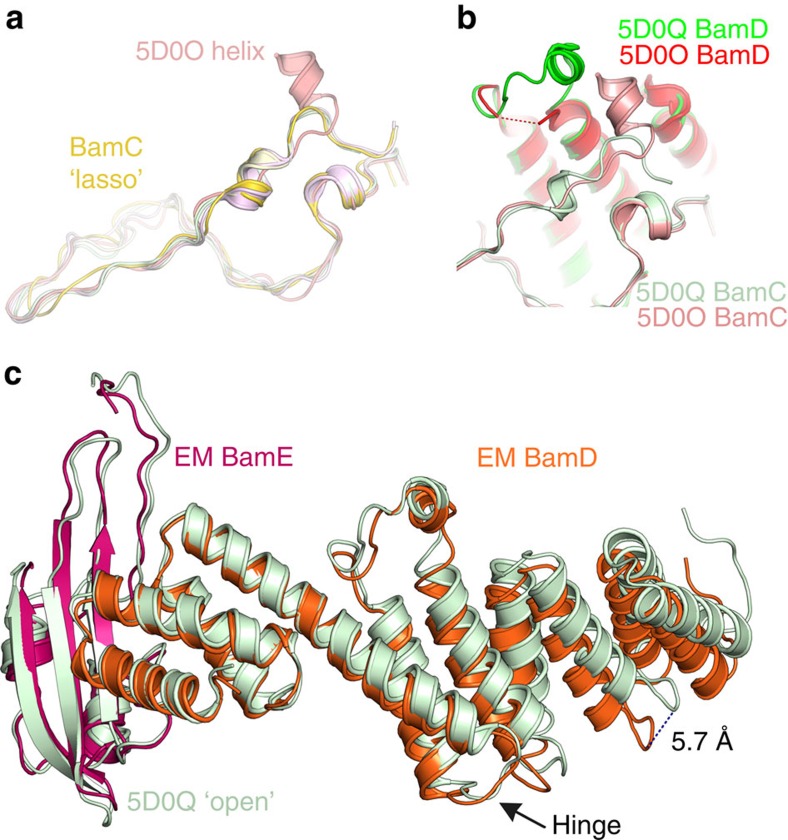
Interactions with the micelle may alter BamD conformation. (**a**) Residues 32–85 in the BamC lasso region are similar in all structures (EM, yellow) except for the ‘lateral closed' structure of intact BAM (5D0O^33^ (pink)), where an extra helical segment is seen. (**b**) This extra helical segment would clash with the 3_10_ helix of BamD (residues 123–132), which is consequently disordered in 5D0O^33^ (red/pink), which is compared with 5D0Q (pale green and green). BamC is pale (pink/green) and BamD is strongly coloured (red/green). (**c**) Conformational change in BamD. BamE of the EM structure is aligned to the equivalent region from the ‘lateral open' BamACDE (5D0Q^33^) structure. The C-terminal half of BamD (left hand side) overlaps almost exactly in the two structures, showing that the interface between BamD and E is preserved. The structures deviate around residue 157 (indicated by black arrow). A helix runs directly from this position to the micelle/membrane. The rest of BamD is displaced as a rigid body by ∼6° at the N terminus.

The N-terminal ‘lasso' of BamC^47^ makes an extensive contact with BamD. The fitted model in the EM structure is similar to this region of BamC observed in previously published ‘lateral open' structures (Fig. 6a). The N-terminal portion of the lasso is in a dramatically different conformation in the ‘lateral closed' structure (5D0O; ref. 33 and Fig. 6), forming a short, amphipathic helical segment that extends towards the postulated position of the membrane. This helical segment would clash with a portion of BamD (residues 123–132) in the EM structure and other crystal structures, which is missing in the crystal structure of the ‘lateral closed' state (Fig. 6b) but present in all of the ‘lateral open' structures.

These observations suggest that BamCD is conformationally dynamic, and in the EM structure we see a possible explanation for this phenomenon. The structures of BamD and BamE are generally similar to previously published structures (Supplementary Table 2), with the exception that BamD appears to have flexed about a hinge in the middle of the molecule. Shown in Fig. 6c are the BamD and BamE structures from the EM model (orange and magenta) and a ‘lateral open' crystal structure (5D0Q), aligned solely on the BamE component (which is essentially identical in both structures; Supplementary Table 2). The interface between BamD and BamE is identical in the two structures, as shown by the superimposition of the C-terminal half of the two BamD molecules. However, midway along BamD, the structures deviate with the N-terminal half of the molecule flexing with respect to the BamDE interface, becoming displaced by around 6 Å. This bending appears to occur as a rigid body around a hinge point in a BamD loop (indicated by an arrow in Fig. 6c), linked by a long α-helix to the hydrophobic helical segment that interacts with the micelle. Comparison of the EM and crystal structures thus suggests a mechanism by which information can be transferred between the protein and the membrane. The interactions between the N-terminal end of BamD and POTRA1 and 2 are different in all structures of the intact (or near intact) complex, whether EM or X-ray, suggesting that this is a dynamic area in the BAM complex (Supplementary Fig. 8).

### The location of BamC

As described above, the N-terminal ‘lasso' of BamC is observed to make extensive interactions with the face of BamD in the cryo-EM structure. However, much weaker density is observed for the N-terminal globular domain of BamC. SDS–PAGE (Fig. 1a) and electrospray ionization–MS (Fig. 1b) confirm the presence of full-length BamC in the material used for the cryo-EM analysis. This suggests that this region of BamC is mobile with respect to the rest of the complex, although interactions are observed with POTRA1 consistent with previous crystal structures^32^,^33^. The C-terminal globular domain is entirely absent from the EM density map, suggesting even greater mobility for this region of BamC.

### Conformational gating is required for BAM function *in vitro*

**Figure 7:**
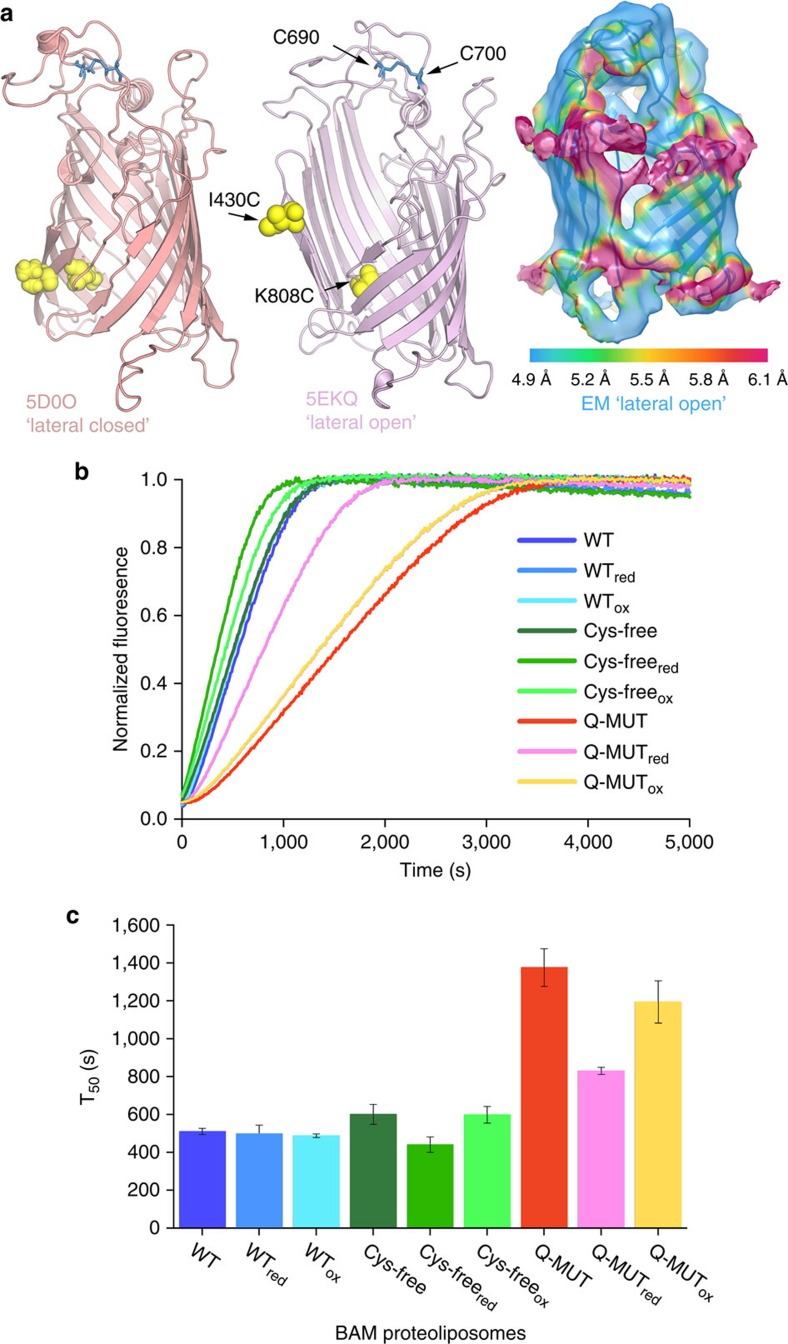
β-Barrel gating of BamA is required for full BAM activity *in vitro*. (**a**) Cys residues introduced into the BamA β-barrel at positions 430 (I430C) and 808 (K808C) (yellow spheres) are hypothesized to be able to form a disulfide in the ‘lateral closed' structure (5D0O^33^) (left image), but not in a ‘lateral open' barrel (for example, 5EKQ^32^) (centre image). The two natural Cys residues (C690 and C700), which were removed, are shown in blue ball and stick. On the right hand side, the EM density is in a similar orientation and coloured according to local resolution^63^, showing that the density around β1–β16 is at a lower resolution and thus more mobile than the body of the barrel. (**b**) Example kinetic traces of OmpT folding measured by its proteolytic activity in the presence of BAM complexes containing wild-type BamA, BamA_C690S/C700S_ (Cys-free) or BamA_C690S/C700S/I430C/K808C_ (Q-MUT; see Supplementary Fig. 1). All experiments were performed with final concentrations of 0.25 μM BAM proteoliposomes, 5 μM OmpT, 1 mM fluorogenic peptide, 35 μM SurA, in oxidizing (1 mM CuSO_4_) or reducing (50 mM dithiothreitol (DTT)) conditions. All experiments were performed in 50 mM glycine-NaOH pH 9.5, 25 °C. (**c**) Bar chart of the average half-time for each folding reaction. These show the mean and s.e.m. from four repeats, across two proteoliposome preparations (see also Supplementary Table 3).

To demonstrate whether lateral gating in the BamA β-barrel is required for BAM-mediated OMP folding activity *in vitro*, as suggested by experiments *in vivo*^33^,^37^, we created a variant designed to constrain the conformation of the barrel. The two endogenous cysteine residues in BamA (C690 and C700) were replaced by Ser (to create the ‘Cys-free' variant BamA_C690S/C700S_; Supplementary Fig. 1d–f) and new Cys residues were introduced on β-strands 1 (I430C) and 16 (K808C) (creating quadruple mutant BamA_C690S/C700S/I430C/K808C_; or ‘Q-MUT'; Supplementary Fig. 1g–i). The latter two amino acid substitutions are predicted to trap BamA in the ‘lateral closed' conformation based on their locations in previous crystal structures^26^ (Fig. 7a) and the variant has been shown to be lethal when substituted for BamA in *E. coli* under oxidizing conditions^37^. Consistent with these *in vivo* observations, proteoliposomes reconstituted with BAM containing Cys-free BamA fold OmpT with similar activity as wild type (Fig. 7b,c). In contrast, proteoliposomes reconstituted with an intact BAM complex containing the Q-MUT BamA are less active than both wild-type BAM or BAM containing Cys-free BamA. This suggests that the disulfide cross-link at C430–C808 does indeed impair, but not prevent, BAM-mediated folding of OmpT *in vitro*. Consistent with this notion, addition of reducing agent rescues activity to near wild-type levels (Fig. 7b,c). Interestingly, the region of the BamA β-barrel that forms the ‘lateral gate', around strands β1 and β16, is among the lowest resolution of any protein component in the EM structure (Fig. 7a), suggesting greater mobility in this part of the molecule, consistent with motion in this part of the structure being important for full activity.

## Discussion

Four crystal structures of the complete, or near-complete, BAM complex have been published recently^32^,^33^,^34^. However, despite this impressive body of work, the mechanism(s) by which BAM assists the folding and membrane insertion of its varied OMP substrates and the roles of individual subunits remain unknown. Although only BamA and BamD are essential in *E. coli*, the BAM complex is most efficient when all five of its subunits are present both *in vivo*^48^ and *in vitro*^13^. Lack of functional BamB has varying effects according to substrate, reducing the levels of many OMPs, but increasing the amount of TolC in the outer membrane^49^. However, despite such substrate-specific effects, defects in OM assembly as a whole are more severe in the absence of BamB than when either BamC or BamE are removed^3^. Despite the important role of BamB in BAM-assisted OMP biogenesis and the proposed role of lateral gating of the BamA β-barrel in the molecular mechanism of BAM, no structures of the intact BAM complex in a ‘lateral open' state have been reported to date. Instead, the ‘lateral open' state has only been observed for BamACDE subcomplexes (that is, in the absence of BamB)^32^, suggesting that dissociation of BamB could be required for lateral gate opening to occur. The cryo-EM structure presented here rules out such a model, showing that the ‘lateral open' conformation of BamA can be formed in an intact BAM complex. In addition, the structure shows that both BamB binding and lateral gate movements modulate the conformation of the BamA POTRA domains. The EM structure presented here confirms, therefore, that both ‘lateral open' and ‘lateral closed' structures can be adopted by the intact BAM complex, presumably representing functional states in the BAM reaction cycle. Indeed, the differences in resolution of POTRA1 and the β1–β16 strands of BamA, together with changes in conformation in BamA and BamD, suggest that these regions may be key factors in OMP folding and OM biogenesis. Whether dynamics in these regions and also in BamC (especially the C-terminal domain) are involved in function and remain dynamic in bilayers remains to be resolved. Techniques such as electron microscopy in lipid nanodiscs^50^ may present further opportunities to examine the structure and dynamics of the membrane embedded proteins.

It has long been proposed that the BamA barrel may open laterally, allowing substrate access to the membrane^51^. Recent crystal structures of detergent-embedded BamA from *Neisseria gonorrhoeae* and *Haemophilus ducreyi* provided the first structural evidence for this hypothesis, revealing remarkably few interactions between the N- and C-terminal β-strands in these β-barrel domains^26^. Molecular dynamics simulations suggest that lateral gate opening can indeed occur, which together with a reduction in the lipid order and membrane thinning adjacent to the barrel's gate, potentially facilitates OMP insertion into the OM^26^. Subsequent experiments in which Cys mutants designed to cross-link the terminal β1 and β16 strands were introduced into BamA *in vivo* were found to be lethal, adding further (albeit indirect) evidence for the barrel-opening mechanism^37^. Here, using the same BamA Cys mutants as described previously^37^ and *in vitro* OMP folding assays, we provide evidence that conformational changes in the BamA β-barrel are required for full BAM function, at least for OmpT. Importantly, OmpT is not able to fold spontaneously into liposomes formed from *E. coli* polar lipids. Furthermore, folding is not facilitated in these liposomes by BamA alone (Supplementary Fig. 3), despite the fact that spontaneous folding of OmpT into synthetic liposomes formed from short-chain (C10) phosphatidylcholine lipids is highly efficient^52^.

The essential lipoprotein BamD makes multiple interactions with the BamA β-barrel and appears to play a role in determining the conformation of the POTRA domains. It makes extensive interactions with POTRA domains 1, 2 and 5, and its removal increases the dynamics of POTRA domains 1 and 2 in molecular dynamics simulations^33^. BamD has been implicated in the recognition of nascent OMPs^8^,^24^, which is also thought to be affected by movements of the POTRA domains^46^. The EM structure presented here builds on this theme, suggesting that BamB may also modulate POTRA conformation (Fig. 4).

The presence of the detergent micelle in the solubilized BAM complex may be a factor influencing the EM structure and, as an analogue of the membrane, allows us to speculate about membrane interactions that may influence BAM structure and function. The short, hydrophobic 3_10_ helix in BamD described above (Figs 5b and 6) interacts with the micelle, with the hydrophobic side chains of residues in the helix sitting in the alkyl chains of the detergent, while the polar residues on either side of the helix are free to interact with the detergent headgroups. The thickness of the micelle at this point (∼40 Å) corresponds well with the thickness of the outer membrane^53^. This sequence of BamD is highly conserved^4^ and the interactions it makes with the micelle (and by inference the membrane) provides a direct link between the membrane and the orientation of the POTRA domains with which BamD interacts. This idea is strengthened by a further interaction with the micelle from BamA POTRA 3 itself (Fig. 5a).

The EM structure suggests that the accessory lipoproteins BamB, C and E may provide additional links between the complex and its membrane environment. They could simply act as additional membrane anchors for the protein. However, the structure raises the possibility that such links could provide a conduit for transfer of information between protein conformation and the lipid environment surrounding the gate in the BamA barrel. This would be consistent with the suggested activity of the BAM complex as a membrane disruptase, allowing substrate binding to effect changes in the lipid environment more widely than simple changes in the BamA barrel conformation. Further structure–function studies on mutant BAM components, particularly in the presence of substrate molecules, will probably shed much light on these ideas and about how this fascinating molecular machine functions to fold and assemble its wide repertoire of OMP substrates in the absence of ATP and a proton motive force.

## Methods

### Expression and purification of BamABCDE mutants

The BamABCDE complex was expressed and purified using a protocol adapted from Roman-Hernandez *et al*.^14^. *E. coli* BL21(DE3) was transformed with plasmid pJH114 (kindly provided by Harris Bernstein^14^) containing all five BAM genes (*BamABCDE-His_6_*) and grown overnight (37 °C, 200 r.p.m.) in LB containing 100 μg ml^−1^ carbenicillin. Cells were diluted 1:100 into fresh TY broth (1 l in a 2 l flask) and grown (37 °C, 200 r.p.m.) to an OD_600_ of 0.5–0.6 before addition of 0.4 mM isopropyl-β-D-thiogalactoside (IPTG) to induce BAM expression. After 1.5 h, cells were harvested with a Beckman JLA-8.1000 rotor (4,000 r.p.m., 15 min, 4 °C) and the pellet resuspended and homogenized in 10 ml l^−1^ 20 mM Tris-HCl pH 8, lysed with a cell disruptor (Constant Cell Disruption Systems, UK), then centrifuged (6,000 *g*, 10 min, 4 °C). The supernatant was ultracentrifuged with a 50.2Ti rotor (45,000 r.p.m., 30 min, 4 °C) to pellet membranes. Pelleted membranes were incubated with 10 ml l^−1^ cold 50 mM Tris-HCl pH 8, 150 mM NaCl, 1% (w/v) DDM at 4 °C for 2 h and the ultracentrifugation repeated to remove insoluble material. Supernatants were then incubated overnight at 4 °C with 2 ml l^−1^ Ni-NTA agarose on a tube roller. Ni-NTA beads were washed with one column volume of 50 mM Tris-HCl pH 8, 150 mM NaCl (TBS), 0.05% (w/v) DDM, 50 mM imidazole and BamABCDE eluted in two column volumes of TBS, 0.05% (w/v) DDM, 500 mM imidazole.

The protein was concentrated to ∼10 mg ml^−1^, using a Vivaspin concentrator (Generon) and gel filtered on a Superdex200, 10/300 GL column running in TBS with 0.05% (w/v) DDM at 0.5 ml min^−1^ and 21 °C. Fractions (0.5 ml) were collected and those containing complete BamABCDE complexes were identified by SDS–PAGE, pooled and concentrated. Protein concentration was determined using the Pierce BCA Protein Assay according to the manufacturer's instructions. The purified BamABCDE complex was concentrated to 13.3 mg ml^−1^ (63 μM), flash frozen in liquid N_2_ and stored at −80 °C.

BamA mutants were prepared using Q5 Site-Directed Mutagenesis (NEB) kit used according to the manufacturer's instructions, to create the Cysteine-free C690S/C700S pseudo-wild type, followed by single rounds of mutagenesis using the pseudo-wild-type gene to add the additional mutations I430C and K808C.

### Reconstitution of the BAM complex into proteoliposomes

Two methods were used to reconstitute purified BAM complex into proteoliposomes. In the first method (‘dilution'), proteoliposomes were made by the method of dilution and ultracentrifugation as described by Roman-Hernandez *et al*.^14^. Briefly, *E. coli* polar phospholipids (Avanti Polar Lipids) were suspended in water at 20 mg ml^−1^, sonicated and 40 μl of the lipid suspension added to 200 μl of purified BamABCDE in TBS containing 0.05% (w/v) DDM at 20 μM. This sample was incubated on ice for 5 min, then diluted with 4 ml of 20 mM Tris-HCl pH 8 and incubated for a further 30 min. Proteoliposomes were pelleted using a Beckman TLA 110 (50,000 r.p.m., 4 °C, 30 min) and resuspended in 200 μl 20 mM Tris-HCl pH 8.

Alternatively, BAM complex was reconstituted into proteoliposomes using a procedure established for the outer-membrane protein FhuA (‘dialysis')^54^. DDM-solubilized BamABCDE (0.3 mg) was mixed with *E. coli* polar lipid films solubilized in 200 μl of TBS+0.05% (w/v) DDM using a 1:0.5 (w/w) ratio of lipid to protein. This was dialysed into detergent-free buffer (20 mM Tris-HCl pH 8, 150 mM KCl, 0.01% (w/v) sodium azide (dialysis buffer)) at 21 °C for 7 days.

To assess whether reconstitution was successful, the samples were centrifuged at 16,000 *g*, the pellet resuspended in dialysis buffer and a sample of the protein–lipid pellet was boiled in SDS-containing loading buffer (50 mM Tris-HCl pH 6.8, 2% (w/v) SDS, 0.1% (w/v) bromophenol blue, 10% (v/v) glycerol) for 10 min, whereas another sample was left unboiled. Samples were then analysed by Tris-Tricine SDS–PAGE. This showed that the full five-protein BAM complex associated with the liposome pellet, and also that BamA was folded, displaying a clear band-shift on boiling. Proteoliposomes created using this procedure resulted in the vast majority of protein incorporated into the liposomes in marked contrast with the yields obtained using the dilution method.

Empty liposomes were made with 220 μl *E. coli* polar lipids solubilized in TBS+0.05% (w/v) DDM. For BamA-proteoliposomes, BamA in 8 M urea, 50 mM glycine-NaOH was folded by ∼30-fold dilution into TBS containing 0.05% (w/v) DDM, 3 M urea and 0.3 mg was then reconstituted by dialysis into proteoliposomes as described for the BAM complex above. The correct folding of BamA was analysed by low SDS–PAGE band-shift assays.

### Low SDS–PAGE analysis

Electrophoresis was carried out on Tris-Tricine buffered SDS–PAGE gels with 30% acrylamide. Where semi-native PAGE was necessary to show heat-modifiability, ‘low SDS' conditions were used, where Tris gel buffer contained no SDS and sample loading buffer contained 0.1% SDS. Loading buffer ( × 2) in this case was 50 mM Tris-HCl pH 6.8, 0.1% (w/v) SDS, 0.1% (w/v) bromophenol blue, 10% (v/v) glycerol. Boiled samples were heated to 100 °C for a minimum 15 min with loading buffer, whereas ‘unboiled' samples were added to sample loading buffer and immediately loaded on the gel. For the semi-native PAGE, the current was 10 mA overnight at 4 °C. Following electrophoresis, gels were stained with Coomassie blue and visualized.

### Purification of BamA and OmpT

BamA and OmpT were purified using a method adapted from McMorran *et al*.^55^. Briefly, the relevant plasmid was transformed into *E. coli* BL21[DE3] cells (Stratagene, UK) and grown in 500 ml lysogeny broth (LB) medium containing 100 μg ml^−1^ carbenicillin at 37 °C with shaking (200 r.p.m.). Expression was induced with 1 mM IPTG when the culture reached an OD_600_ of 0.5–0.6 and then harvested after 4 h by centrifugation (5,000 *g*, 15 min, 4 °C). Cells were resuspended in 20 ml 50 mM Tris-HCl pH 8.0, 5 mM EDTA, 1 mM phenylmethylsulfonyl fluoride, 2 mM benzamidine and lysed by sonication (6 × 1 min bursts with 1 min cooling on ice between each sonication). The insoluble fraction was collected by centrifugation (25,000 *g*, 30 min, 4 °C), resuspended in 20 ml 50 mM Tris-HCl pH 8.0, 2% (v/v) Triton X-100 and incubated for 1 h at room temperature, with gentle agitation. The insoluble fraction was again pelleted (25,000 *g*, 30 min, 4 °C) and the inclusion bodies washed twice by resuspending in 50 mM Tris-HCl pH 8.0, incubating for 1 h at room temperature with gentle agitation, followed by centrifugation (25,000 *g*, 30 min, 4 °C). The inclusion bodies were solubilized in 25 mM Tris-HCl, 6 M Guanidine-HCl pH 8.0 and centrifuged (20,000 *g*, 20 min, 4 °C). The supernatant was filtered (0.2 μM polyvinylidene difluoride syringe filter, Sartorius, UK) and purified further by gel filtration using a Superdex 75 HiLoad 26/60 column (GE Healthcare) (OmpT) or Sephacryl-S200 (GE Healthcare) (BamA), equilibrated with 25 mM Tris-HCl, 6 M Gdn-HCl pH 8.0. Peak fractions were concentrated to ∼500 μM using Vivaspin 20 (5 kDa MWCO) concentrators (Sartorius, UK), snap-frozen in liquid nitrogen and stored at −80 °C.

### Expression and purification of His-tagged SurA

His-tagged SurA was expressed and purified using a protocol adapted from Burmann *et al*.^56^. The pET28b plasmid, containing the mature *SurA* gene with an N-terminal, 6 × His-tag and thrombin cleavage site, was transformed into BL21[DE3]pLysS cells (Stratagene). Cells were grown in LB medium containing 30 μg ml^−1^ kanamycin at 37 °C with shaking (200 r.p.m.) until the culture reached an OD_600_ of ∼0.6. The temperature was then lowered to 20 °C and expression induced with 0.4 mM IPTG. Following overnight expression (∼18 h) cells were harvested by centrifugation, resuspended in 25 mM Tris-HCl pH 7.2, 150 mM NaCl, 20 mM imidazole, containing EDTA-free protease inhibitors (Roche) and lysed using a cell disrupter (Constant Cell Disruption Systems, UK). Following centrifugation to remove cell debris (20 min, 4 °C, 39,000 *g*), the lysate was applied to 5 ml HisTrap columns (GE Healthcare) and washed with 25 mM Tris-HCl pH 7.2, 150 mM NaCl and 20 mM imidazole. His-tagged SurA was denatured on-column with 25 mM Tris-HCl, 6 M Guanidine-HCl pH 7.2 and eluted with a gradient of 25 mM Tris-HCl, 6 M Guanidine-HCl pH 7.2 and 500 mM imidazole. Fractions containing SurA were pooled and the protein refolded by dialysis against 50 mM glycine-NaOH pH 9.5. Refolded HT-SurA was concentrated to ∼200 μM using Vivaspin 20 (5 kDa MWCO) concentrators (Sartorius, UK), aliquoted, snap-frozen in liquid nitrogen and stored at −80 °C.

### BAM activity assays

#### Activity assays of BAM reconstituted by different methods

For BAM activity measurements in proteoliposomes formed by dilution, resuspended proteoliposomes were diluted two-fold into 50 mM glycine-NaOH pH 9.5 containing 2 mM of the fluoropeptide Abz-Ala-Arg-Arg-Ala-Tyr(NO_2_)-NH_2_ (Peptide Synthetics). OmpT and SurA were then mixed to form a solution with final concentrations of 20 μM OmpT, 140 μM His_6_-SurA in 50 mM glycine-NaOH pH 9.5 and 1.75 M urea. This sample (SurA-OmpT ‘subreaction') was then immediately diluted two-fold into proteoliposome-containing solutions to initiate the folding reaction. All OmpT folding reactions were carried out in 30 μl final reaction volume.

For BAM activity measurements in proteoliposomes formed by dialysis, the proteoliposomes were pelleted by centrifugation (16,000 *g*, as above). The pellet was then resuspended in dialysis buffer and diluted to a concentration of 5 μM in 50 mM glycine-NaOH pH 9.5 containing 2 mM of the fluoropeptide Abz-Ala-Arg-Arg-Ala-Tyr(NO_2_)-NH_2_ (Peptide Synthetics). This was then mixed with the same SurA-OmpT subreactions as described above. Fluorescence emission following excitation at 325 nm was monitored at 430 nm with readings every 10 s for up to 5 h using a Clariostar plate reader (BMG Labtech GmbH). The signal was normalized following subtraction of the average background signal produced at the zero time point.

#### Activity assays of wild-type and mutant BAM complexes

In experiments to compare the activity of wild-type and mutant BAM complexes, the protein concentration of proteoliposomes prepared by extensive dialysis was determined by the Pierce BCA Protein Assay and diluted to a constant 3.5 μM. BAM complex proteoliposomes (0.5 μM) were incubated briefly with 2 mM of the fluoropeptide Abz-Ala-Arg-Arg-Ala-Tyr(NO_2_)-NH_2_ (Peptide Synthetics). Purified OmpT denatured in 8 M urea, 50 mM glycine-NaOH at 100 μM was diluted ten-fold into solutions of 70 μM His_6_-SurA in folding buffer (50 mM glycine-NaOH pH 9.5). These SurA-OmpT solutions were mixed and instantly diluted two-fold into the proteoliposome-containing solutions. The final concentrations of the reaction components were 5 μM OmpT, 35 μM SurA, 0.25 μM BAM complex and 1 mM fluorogenic peptide. Assays were then performed as described above. To calculate half-times, a baseline was fitted to the region of the trace where fluorescence increases were no longer observed.

### Non-covalent MS

Purification of BamABCDE for non-covalent MS was performed using a modified version of the above procedure, as outlined above. Modifications included using LB rather than tryptone yeast (TY) broth for growth and elution of purified BamABCDE from Ni-NTA agarose was performed using TBS with 0.5% (v/v) Triton X-100. Gel filtration was carried out as above in TBS with 0.5% (v/v) Triton X-100.

The purified BAM complex was buffer exchanged into 200 mM ammonium acetate, 0.02% (v/v) Triton X-100 pH 6.9 using Zeba spin desalting columns (Thermo Scientific, UK) immediately before MS analysis. Nanoelectrospray ionization–MS spectra were acquired using a Synapt HDMS hybrid quadrupole–travelling wave–time-of-fight mass spectrometer (Waters Corporation, UK) using in-house prepared platinum/gold-coated borosilicate capillaries. Typical instrument parameters included the following: capillary voltage 1.2–1.6 kV, cone voltage 120 V, trap collision voltage 60 V, transfer collision voltage 10 V, trap DC bias 20 V and backing pressure 7 mBar. Data were processed using MassLynx v4.1, (Waters Corporation, UK) and UniDec^57^.

### Denaturing MS for molecular mass determination

Purified BAM complex was precipitated by chloroform–methanol to minimize detergent contamination. Briefly, a sample of the BAM complex in TBS, 0.05% (w/v) DDM (50 μl, 10 μM) was taken, and methanol (150 μl) and chloroform (50 μl) were added. The solution was mixed by vortexing, water (100 μl) was then added and the solution was mixed again before centrifuging (10,000 *g*, 2 min). The upper aqueous phase was removed (leaving the white protein pellet and the lower organic phase) and methanol (150 μl) was then added. The solution was mixed by vortexing, centrifuged (10,000 *g*, 2 min) and the supernatant removed. The precipitated protein was air dried in a laminar flow hood. The dried protein pellet was resuspended in formic acid (4 μl) and 18 MΩ H_2_O (Purite) was then added (46 μl) for subsequent MS analyses.

Proteins were analysed intact using online desalting liquid chromatography–MS on a nanoAcquity LC system interfaced to a Xevo G2-S mass spectrometer (Waters Ltd., Wilmslow, Manchester, UK). The sample (2 μl) was loaded onto a MassPREP protein desalting column (Waters Ltd, Wilmslow, Manchester, UK), which was washed with 2% (v/v) solvent B in solvent A (solvent A was 0.1% (v/v) formic acid in water, solvent B was 0.1% (v/v) formic acid in acetonitrile) for 5 min at 40 μl min^−1^. After valve switching, the bound proteins were eluted using a fast gradient of 2-40% (v/v) solvent B in A over 1 min at 0.5 μl min^−1^. The column was subsequently washed with 95% (v/v) solvent B in A for 6 min and re-equilibrated with 5% (v/v) solvent B in solvent A for the next injection. The column eluent was infused into a Xevo G2-S mass spectrometer (Waters Ltd, Wilmslow, Manchester, UK). Data were processed using MassLynx v4.1, (Waters Corporation, UK) and UniDec^57^.

### Electron microscopy

Purified BAM complex in TBS with 0.05% (w/v*) n*-dodecyl β-D-maltopyranoside (DDM) at a concentration of 13 mg ml^−1^ was diluted 1:2 in the same buffer. A 4 μl aliquot of solution was applied to a quantifoil r3.5/1 EM grid (Quantifoil Micro Tools), allowed to incubate at room temperature and 95% humidity for 30 s, then manually blotted with a Whatman filter paper. A second 4 μl aliquot of BAM solution was added and the grid blotted and vitrified by plunging into liquid ethane using a Leica EM GP plunge freezer (Leica Microsystems).

The grids were imaged on a Titan Krios electron microscope (FEI Corporation) operating at 300 kV. Seven thousand and five hundred micrographs with a nominal defocus range of 1.5–3.5 μM were recorded with a Gatan K2 Summit energy-filtered direct detector (Gatan, Inc.) as 20 frame movies with a total electron dose of ∼40 *e*^−^ Å^−2^. The sampling rate was 1.04 Å per pixel.

### Image processing

Individual micrograph frames were combined into stacks using IMOD^58^ and drift corrected using Motioncorr^59^. Individual particles were windowed manually using EMAN2 (ref. 60) and automatically using Relion^61^. The raw particle set (472,857 particles) was culled through repeated cycles of two- and three-dimensional classification using Relion^43^. The vast majority of these discarded particles were incorrectly picked by the automatic particle picking algorithms required to deal with a data set consisting of ∼7,200 individual micrographs. These ‘junk particles' represented carbon, ice contamination and a small amount of aggregated protein material. A total of ∼120,000 particles were identified as BAM and after further classification, a total of 95,878 particles were used for structural analysis. Following alignment and three-dimensional reconstruction, the final structure was generated after performing per-particle motion correction on individual micrograph frames^62^ and post-processed using Relion. Frames 4–14 of the original micrographs were used for the final reconstruction, for a total dose of 20 *e*^−^ Å^−2^. The local resolution of the final map was calculated using resmap^63^ and it was then filtered by local resolution using LocalFilt (in press, Chris Ayett, ETH-Zurich personal communication). The final resolution was determined by ‘gold standard' Fourier shell correlation of two independent half maps (Supplementary Fig. 4).

### Atomic model fitting

An initial model of the BAM–ABCDE complex was constructed by manually fitting BAM subunits from previously published crystal structures to the EM density using UCSF Chimera^64^. BamA was constructed from 5D0Q^33^ and 5D0O^33^. BamC and BamD were taken from 5D0Q^33^, BamE from 5D0O^33^ and BamB constructed from 3Q7O^25^ and 2YH3^24^. Selenomethionines in each model were replaced with methionine. The initial model was then refined by flexible fitting to the EM density using MDFF^44^ and Monte Carlo simulations with RosettaEM^45^.

### Analysis of angles between BamA POTRA domains

To calculate angles between *E. coli* BamA POTRA domains in all available structures, an arbitrary point within the loop on either side of each POTRA domain was identified. These same points in all X-ray and EM structures were the Cα atoms of F24, T93, A175, Y266, N345 and R421. The angles between each pair of POTRA domains were then calculated by placing three of these points on a plane. For example, the angle between POTRA 2 and 3 (Fig. 4a main text) was calculated as the in-plane angle for T93-A175-Y266, the three spheres shown in the figure. This is 120° for the EM structure.

### Data availability

The EM density map and fitted model have been deposited in the Protein Data Bank under accession code EMDB EM-4061 and PDB 5LJO, respectively. All data that support the findings of this study are available from the corresponding author upon request.

## Additional information

**How to cite this article**: Iadanza, M. G. *et al*. Lateral opening in the intact β-barrel assembly machinery captured by cryo-EM. *Nat. Commun.* 7:12865 doi: 10.1038/ncomms12865 (2016).

## Supplementary Material

Supplementary Information Supplementary Figures 1-10, Supplementary Tables 1-3 and Supplementary References
