# Supplementary material for: Lateral opening in the intact β-barrel assembly machinery captured by cryo-EM
Source: Nat Commun. 2016 Sep 30;7:12865. doi: 10.1038/ncomms12865 (PMC5056442; doi:10.1038/ncomms12865)
Supplement: Supplementary Information — Supplementary Figures 1-10, Supplementary Tables 1-3 and Supplementary References [file ncomms12865-s1.pdf]

Lateral opening in the intact β-barrel assembly machinery captured by cryo-EM

Iadanza et al.

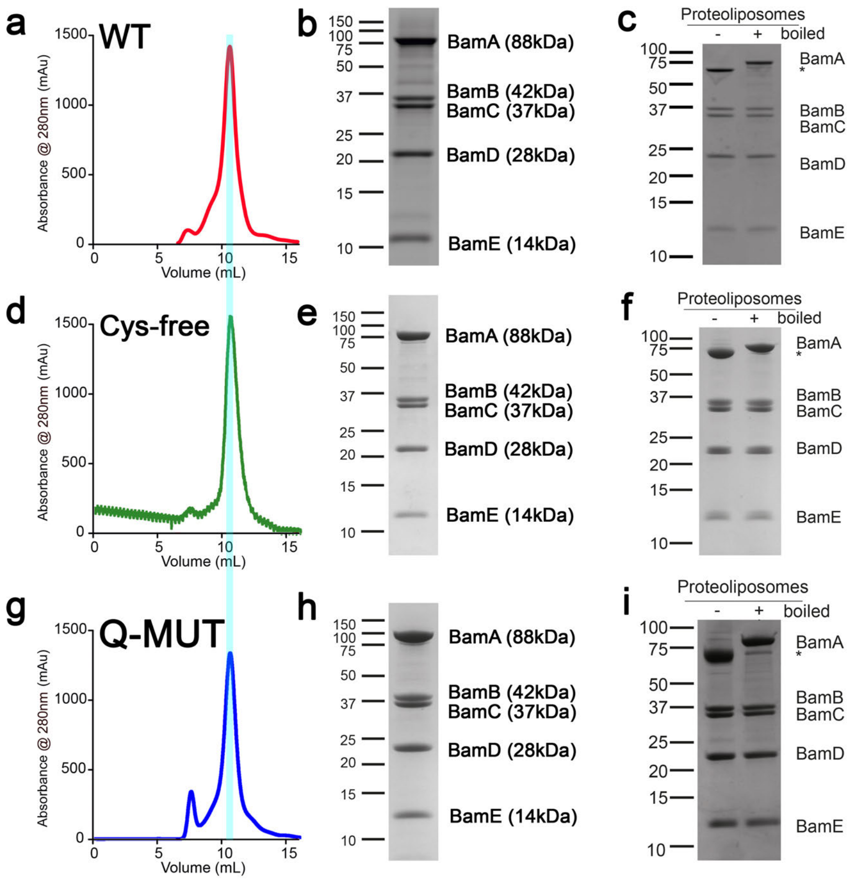

Supplementary Figure 1

Supplementary Figure 1. Purification of the full BamABCDE complex and complexes containing BamA mutants**.** (a,d,g) The final step in BAM complex purification was size exclusion chromatography using an analytical S-200 column. An example trace is shown, with protein eluting from the column as a single peak. (b,e,h) Elution fractions from this peak (indicated by the transparent cyan bar) were analyzed by SDS-PAGE confirming the presence of all five BAM subunits. Gels show the peak fractions that were pooled, concentrated and used for structural analysis and reconstitution into liposomes. (c,f,i) Reconstitution of the BAM complexes into liposomes formed from *E.coli* polar lipids. The unboiled and boiled sample shows a band-shift for BamA, consistent with the protein being folded in the proteoliposomes. Molecular weight markers are in kDa.

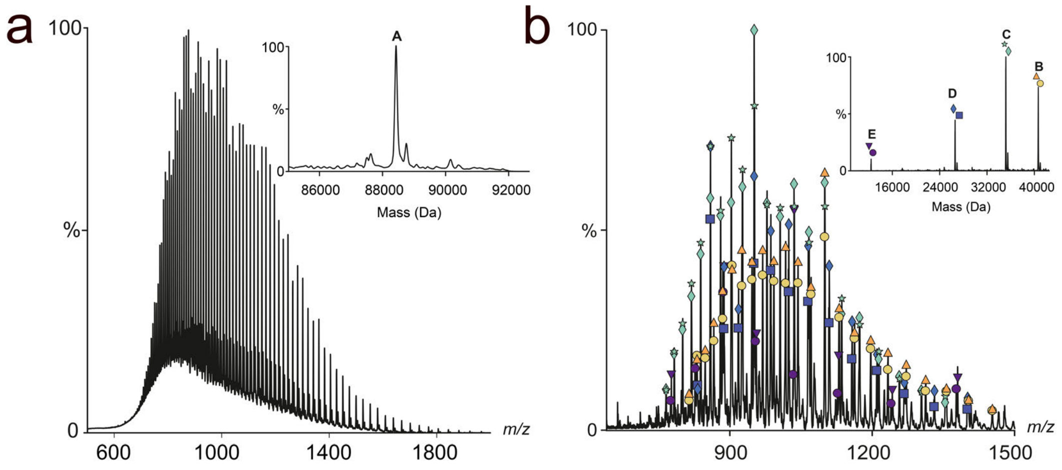

c

Protein	Theoretical mass (Da)	Measured mass (Da)	(Measured-Theoretical) (Da)	PTM
BamA	**88426**	88426	0	-
BamB	**39853**	40643	790	3x C_{16}
		40663	810	2x C_{16} 1x C_{18}
BamC	**34371**	35163	792	3x C_{16}
		35183	812	2x C_{16} 1x C_{18}
BamD	**25790**	26583	793	3x C_{16}
		26603	813	2x C_{16} 1x C_{18}
BamE	**11596**	12383	787	3x C_{16}
		12413	817	2x C_{16} 1x C_{18}

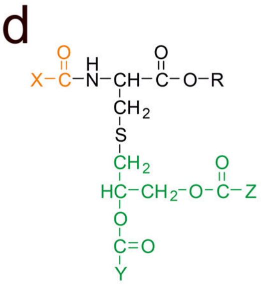

Supplementary Figure 2

Supplementary Figure 2. Denaturing mass spectrometry for molecular weight determination of BAM subunits. (a) Denatured mass spectrum of BamA. (b) Denatured mass spectrum of BamB, C, D and E. The insets in (a) and (b) show the deconvoluted spectra. (c) Theoretical and measured masses. The mass discrepancy between the predicted and experimentally measured values is shown, along with the post-translational modification (PTM) associated with the observed mass difference. Note that for all subunits with PTMs, two species are observed with different lipid anchors. The number of double bonds in the acyl chains of the acyl chains cannot be determined at this resolution. (d) Chemical structure of the N-terminal modified Cys residue. X,Y, and Z denote the position of the PTMs indicated in part (c).

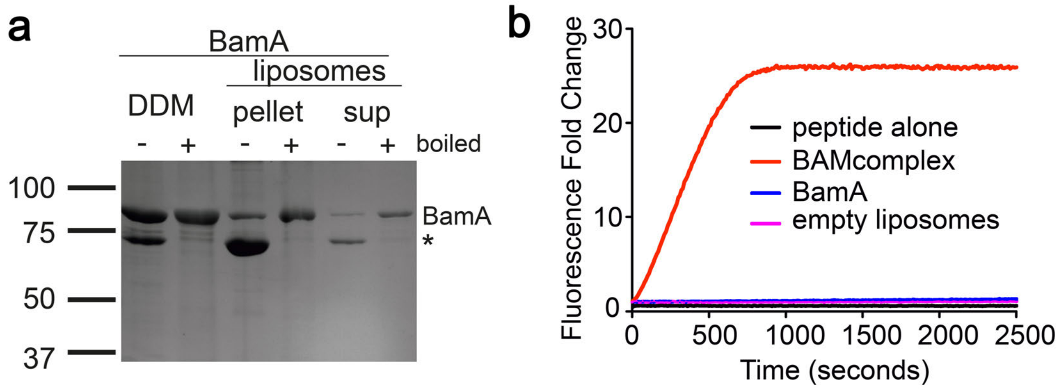

Supplementary Figure 3

Supplementary Figure 3. To determine whether BamA reconstituted in proteoliposomes possesses the catalytic activity of the intact BAM complex in assisting OmpT folding, the following control was carried out. Isolated BamA was purified in an unfolded form from inclusion bodies (see Methods). Purified BamA was folded in TBS containing 0.05% (w/v) DDM and 3 M urea. The same dialysis procedure for proteoliposome production was followed as for the whole BAM complex, after which proteoliposomes were pelleted and resuspended. (a) Semi-native PAGE demonstrates a band-shift of folded BamA relative to the unfolded protein. BamA in detergent ('DDM' lanes) is ~40% folded. In addition, BamA is present in the proteoliposome pellet ('pellet' lanes), indicating it is associated with the liposomes, and shows similar levels of folding. (b) Activity of this BamA proteoliposome sample is compared to that of the BAM complex sample, using the OmpT reporter assay. The catalytic activity of the full BAM complex is measurable as assisting OmpT folding leads to cleavage of a fluorogenic peptide over a short timescale. Proteoliposomes containing only BamA are unable to assist OmpT folding, and show no significant change in fluorescence from empty proteoliposomes.

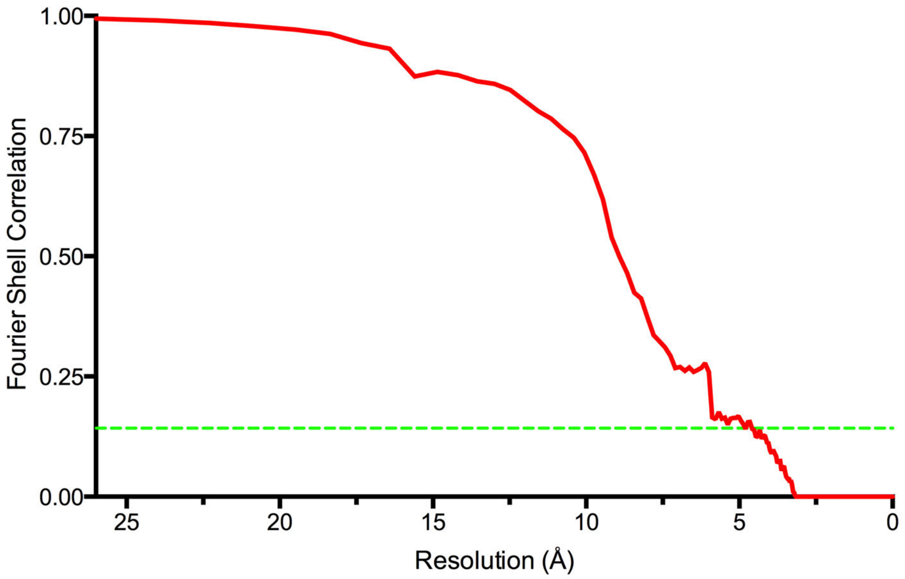

Supplementary Figure 4

Supplementary Figure 4. Fourier shell correlation (FSC) of the EM density. The FSC was calculated from two independent half-maps and resolution quoted at the 0.143 threshold.

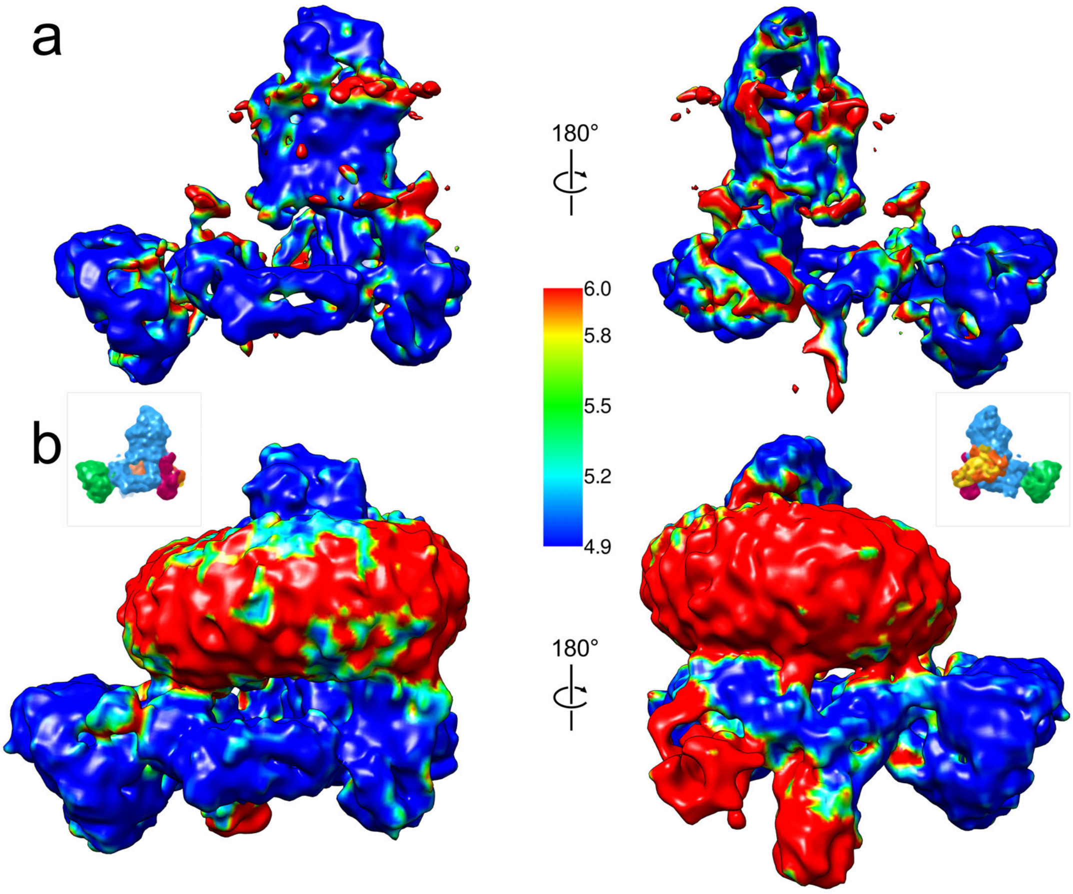

Supplementary Figure 5

Supplementary Figure 5. Local resolution estimates of the cryo-EM structure. The BAM complex EM structure at high (a) and low (b) threshold values, colored by local resolution as shown in the accompanying key. Poorer resolution is observed in regions of the protein that are less well resolved due to mobility (POTRA1, BamC N-terminal globular domain) and in the detergent micelle. Local resolution values were calculated using ResMap[12]. Thumbnails are colored as in Fig. 2 in the main text.

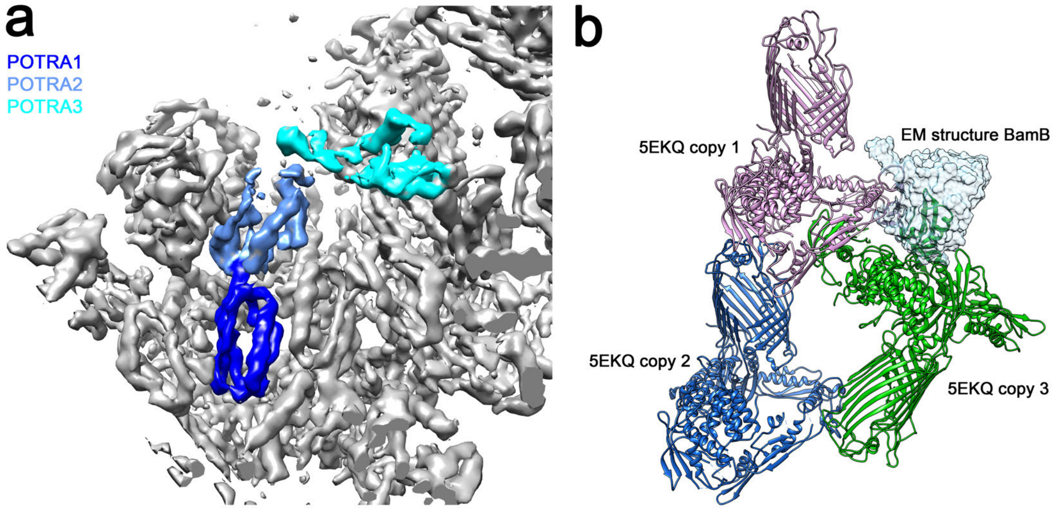

Supplementary Figure 6

Supplementary Figure 6. Packing interactions in crystals of the BAM complex. (a) BamA POTRA 1 (dark blue), POTRA 2 (light blue), and POTRA 3 (cyan) in the crystal structure of a lateral open, BamACDE (i.e. BamB-less) crystal structure (5DOQ[14]) are nested in a deep cleft formed by the POTRAs and BamD of neighboring molecules in the crystal lattice, restricting their mobility and/or position. (b) Three adjacent molecules in a different laterally open BamACDE complex, (i.e. again, BamB-less). The atomic model is shown using a cartoon representation. The pseudo atomic model for the entire cryo-EM structure was aligned to the pink BamACDE complex in this view, and the corresponding position of the EM structure for BamB is shown as a transparent surface. POTRA1 intrudes into this space making the presence of BamB incompatible with this crystal packing arrangement.

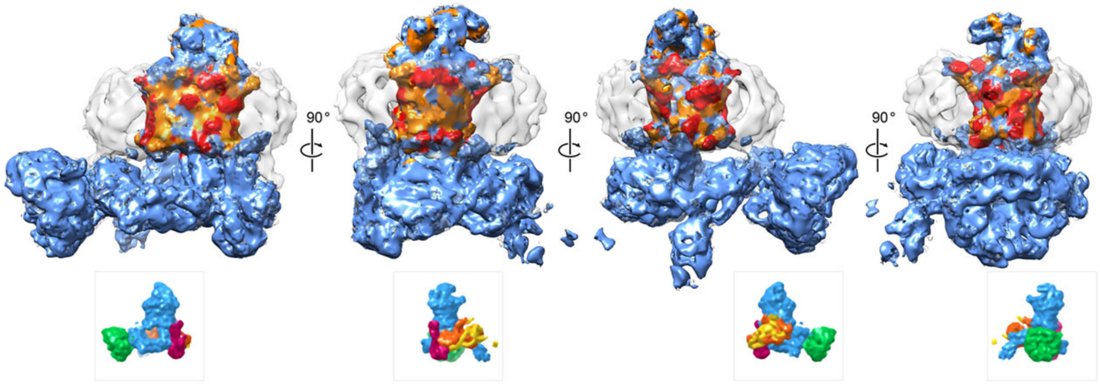

Supplementary Figure 7

Supplementary Figure 7. The hydrophobic residues of the BamA β-barrel are buried in the detergent micelle. The EM-BAM structure is shown (in blue) with the the aromatic residues (Trp, Tyr, and Phe) colored red and other hydrophobic residues of the BamA barrel (Gly, Pro, Ile, Leu, Val and Met) colored orange. The detergent micelle (transparent) shields these residues in a manner consistent with their proposed position with the native lipid environment for the BAM complex. Thumbnails are colored as in Fig. 2 in the main text.

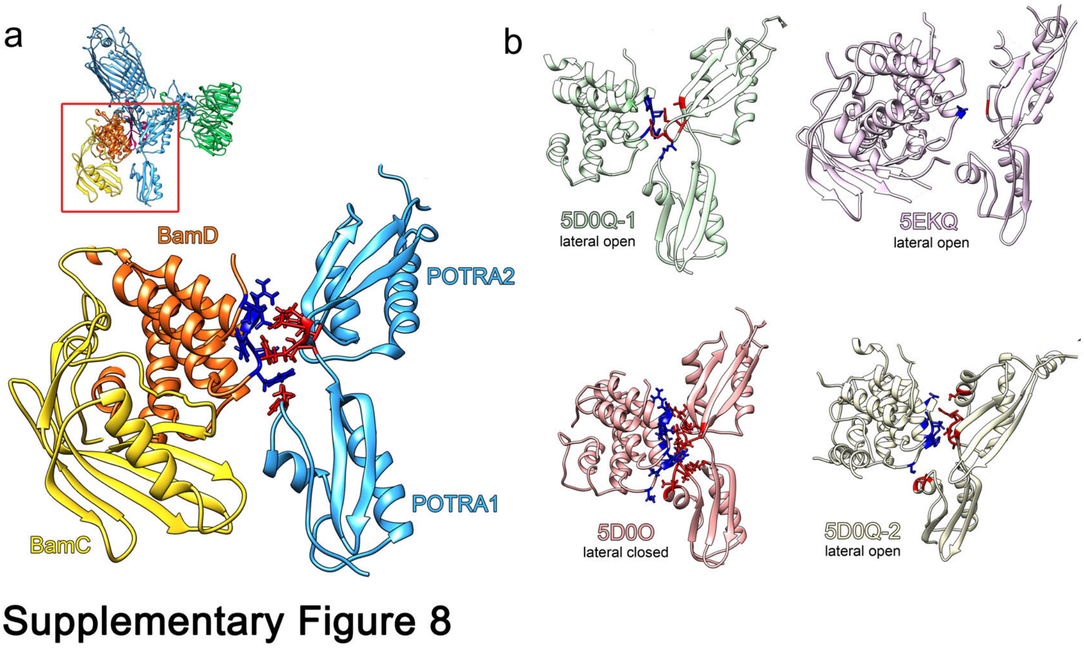

Supplementary Figure 8: Contacts between BamA POTRA domains 1 and 2 and BamD. (a) Amino acid residues from POTRA domains 1 and 2 (red) and BamD (blue) predicted to make contacts in the EM structure. (b) POTRA domains 1 and 2 (red) and BamD (blue) residues predicted to make contacts in three laterally open (5D0O[14], 5EKQ[20]) and one laterally closed (5D0O[14]) crystal structures.

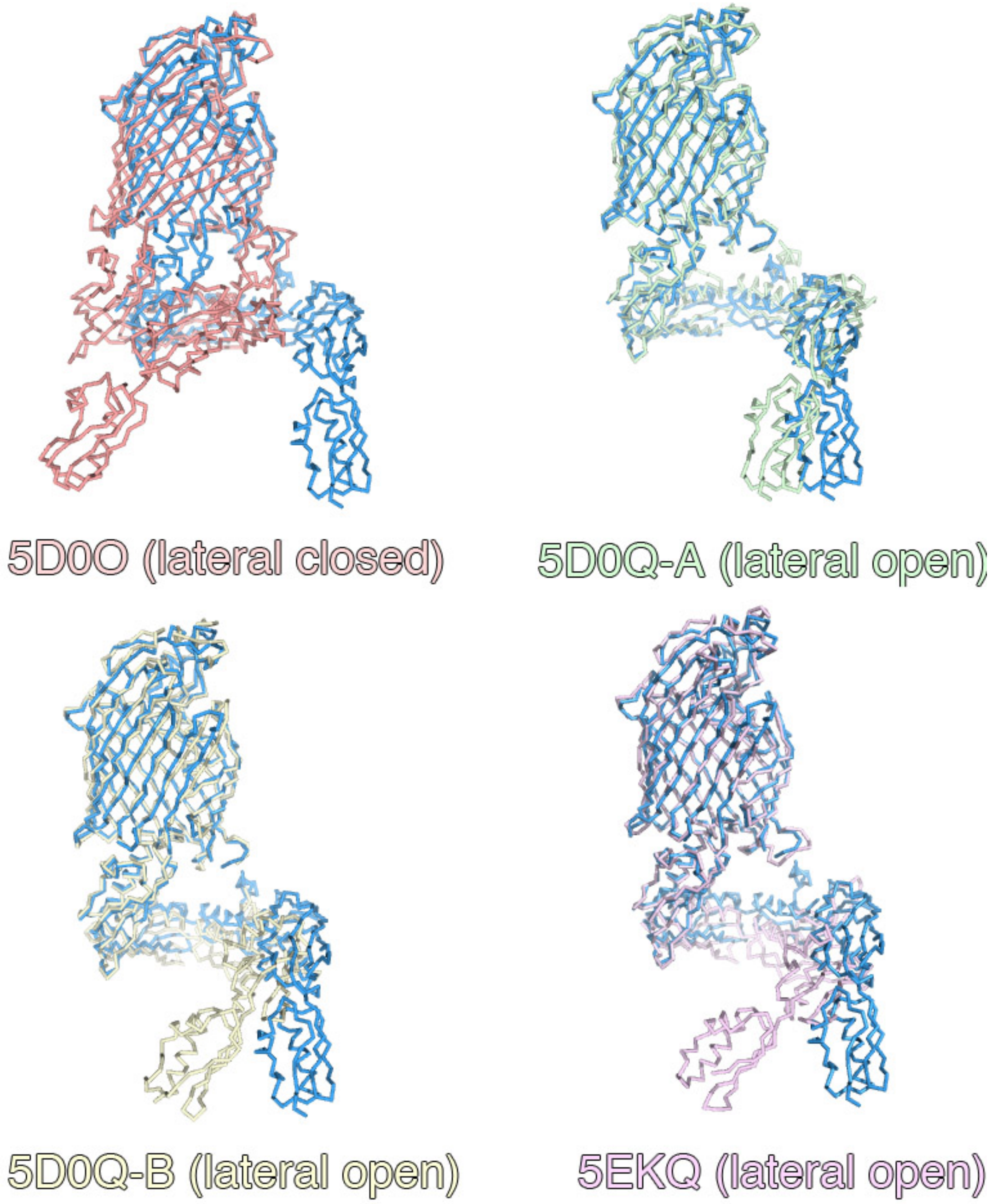

Supplementary Figure 9

Supplementary Figure 9: Comparison of BamA structure. BamA from the laterally open EM derived model (blue) compared to a laterally closed structure (5D0O[14], red) and three laterally open structures 5D0Q-A[14] (green), 5D0Q-B[14] (yellow), and 5EKQ[20] (pink).

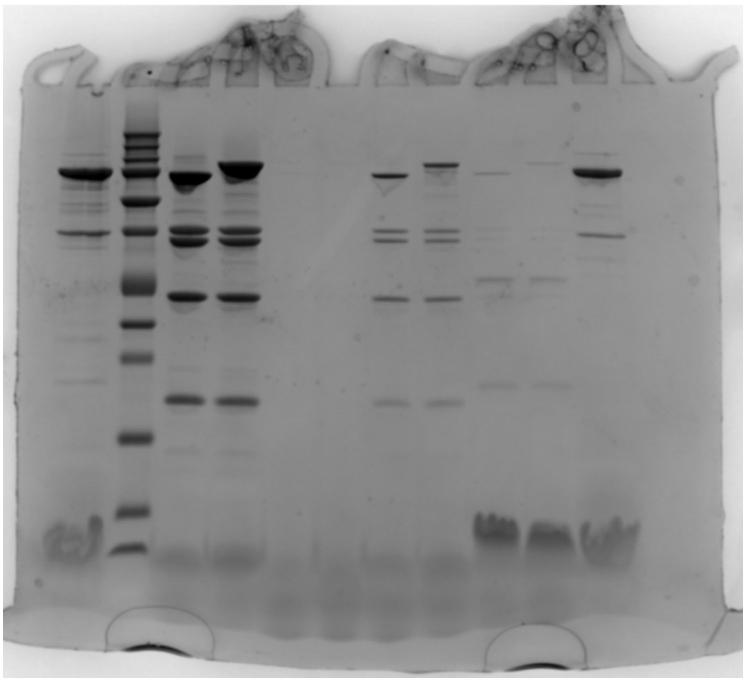
A

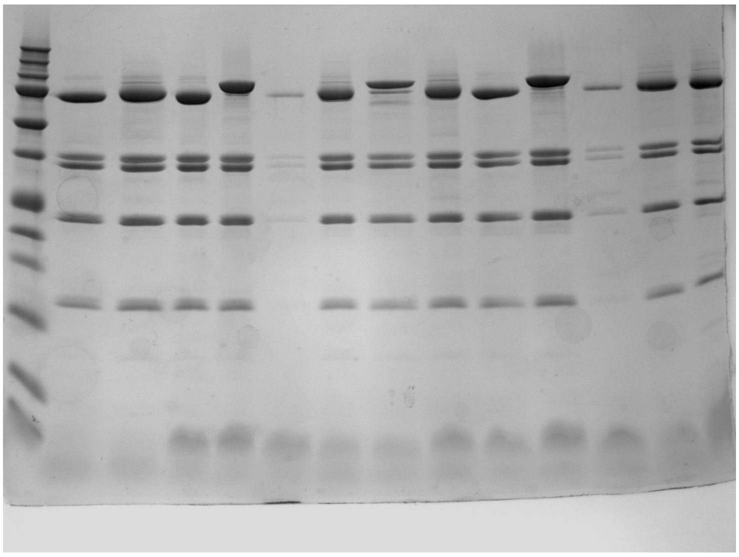
B

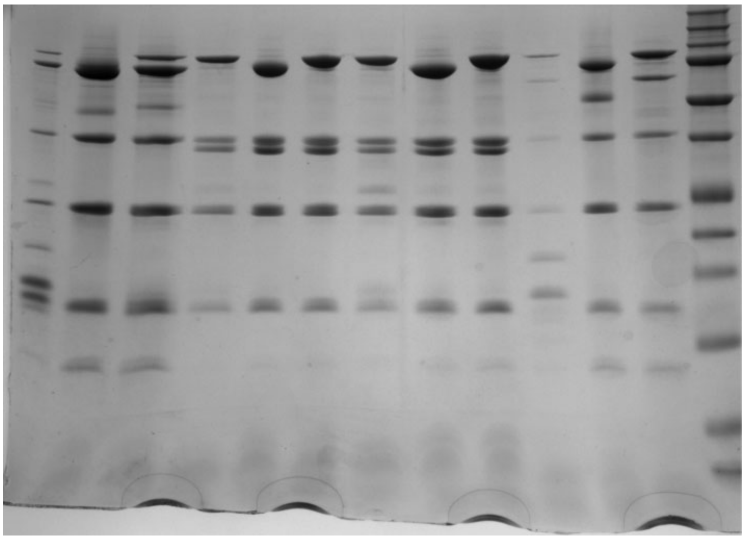
C

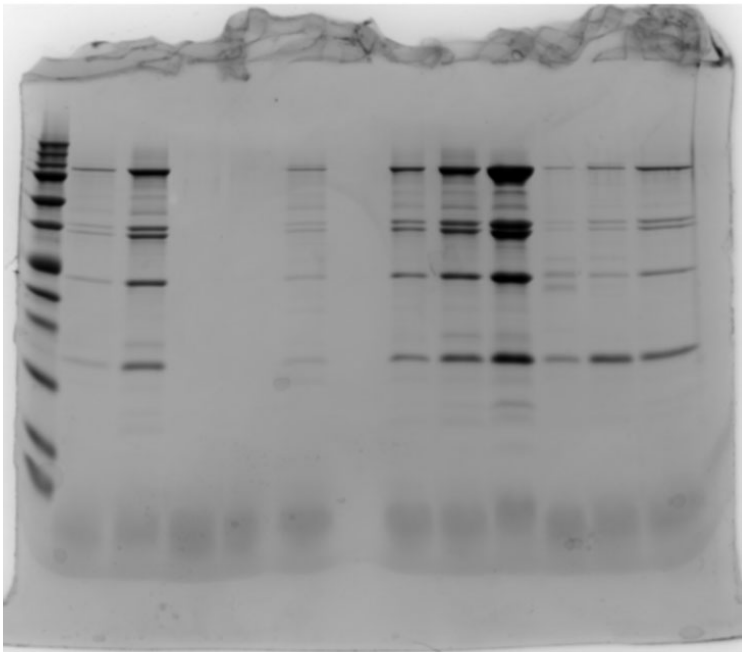

D

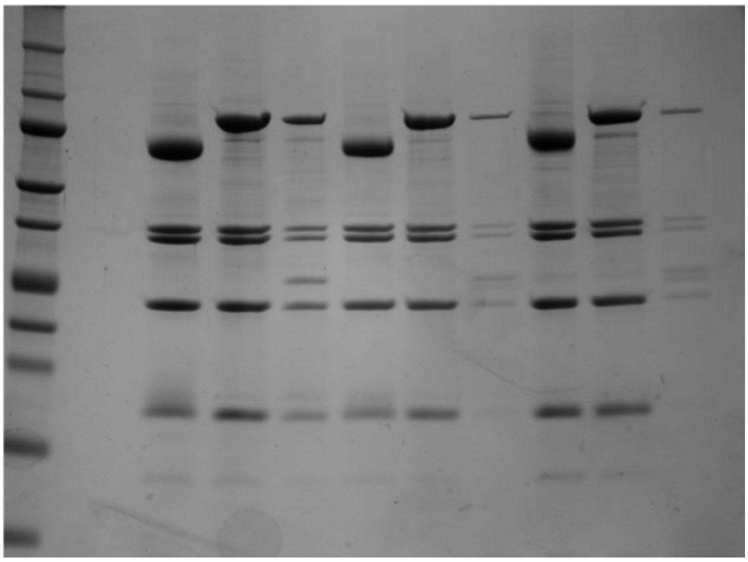

E

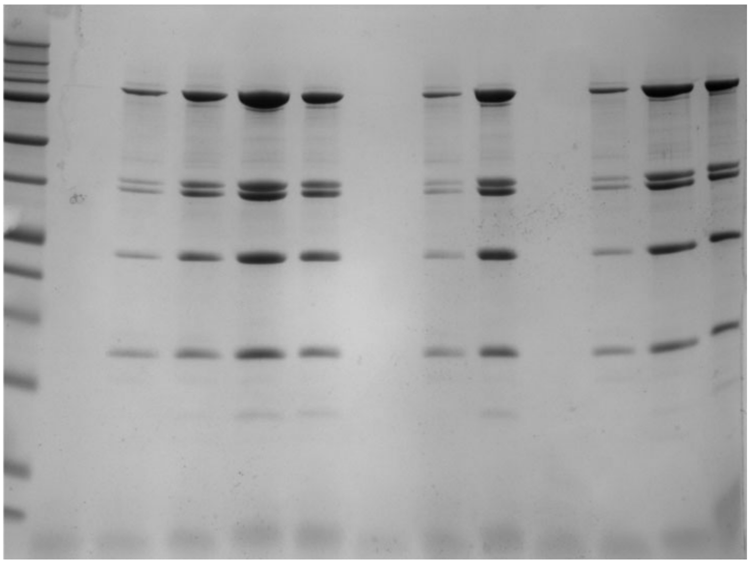

F

G

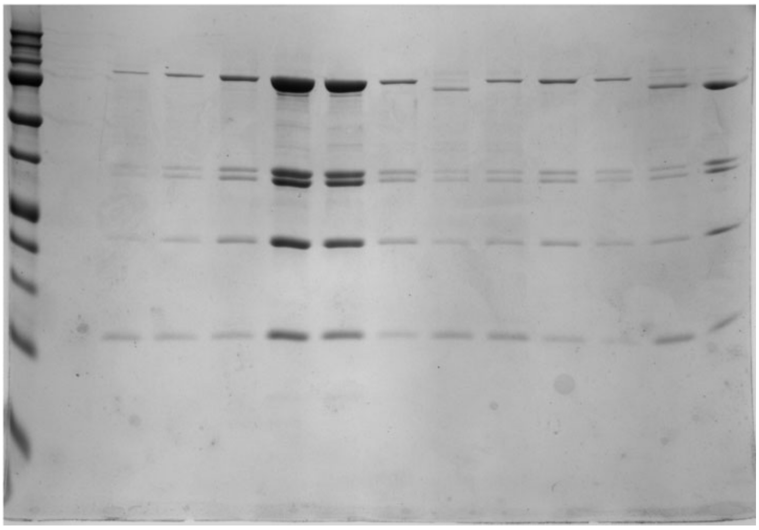

H

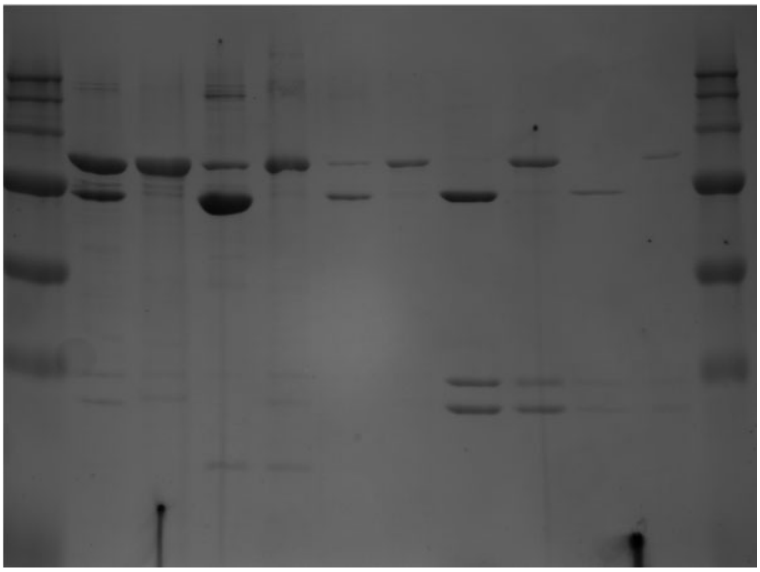

Supplementary Figure 10: Full gels from main and supplementary figures. (A) Lanes 7 and 8 appear in Figure 1 and Supplementary Fig. 1c (B) Lane 2 appears in Figure 1. (C) Lanes 5 and 6 appear in Supplementary Fig. 1f (D) Lane 3 appears in Supplementary Fig. 1b. (E) Lanes 9 and 10 appear in Supplementary Fig. 1i. (F) Lane 5 appears in Supplementary Fig. 1h. (G) Lane 7 appears in Supplementary Fig. 1e. (H) Lanes 2-7 appear in Supplementary Fig. 3. All lanes are counted from the left.

Supplementary Table 1. Native mass spectrometry of the BAM complex. Theoretical and experimentally determined masses of the BAM complex and observed subcomplexes.

	Theoretical (Da)	Experimental (Da	Mass Error (Da	Mass Error (%)
AB	129069	129184 ± 10	115	0.09
ACDE	162555	162785 ± 25	230	0.14
ABCDE	203218	203456 ± 22	238	0.12
$ABCDE_2$	215601	216043 ± 21	442	0.21

*Theoretical masses are calculated from the measured masses of the subunits (Supplementary Fig. 3). For consistency, the post-translational modifications with the lowest mass were used for calculations.

Supplementary Table 2. Structural similarity between crystallographic and EM BAM complex structures.

		5D0O	5D0Q-#1	5D0Q-#2	5EQK
EM structure	Barrel	5.092	1.627	1.773	2.006
	POTRAS	7.15	4.5	6.46	7.612
	B	2.319	-	-	-
	C lasso	5.298	2.47	7.192	2.686
	D	1.386	1.156	1.096	1.216
	E	1.619	1.466	1.448	1.762
5D0O	Barrel	0	5.005	5.068	4.78
	POTRAS	0	5.703	4.58	5.395
	C lasso	0	4.522	4.504	4.31
	D	0	1.199	1.161	1.292
	E	0	1.156	1.091	0.763
5D0Q-#1	Barrel	5.005	0	0.887	1.446
	POTRAS	5.703	0	4.635	4.85
	C lasso	4.522	0	0.785	1.149
	D	1.199	0	0.235	1.244
	E	1.156	0	0.336	1.232
5D0Q-#2	Barrel	5.068	0.887	0	1.627
	POTRAS	4.58	4.635	0	2.206
	C lasso	4.504	0.785	0	1.229
	D	1.161	0.235	0	1.147
	E	1.091	0.336	0	1.174
5EQK	Barrel	4.78	1.446	1.412	0
	POTRAS	5.395	4.85	2.206	0
	C lasso	4.31	1.149	1.229	0
	D	1.292	1.244	1.147	0
	E	0.763	1.232	1.174	0

RMSD (Å) of in common Cα atoms for each subunit of the BAM complex in the cyro-EM structure and four previously published structures of the Bam-ABCDE or Bam-ACDE complex. The β-barrel and POTRA domains are considered separately. RMSD calculations were performed on residues 32-85 of BamC, the 'lasso' that makes contact with BamD excluding residues 25-31, which are poorly modelled in many structures. As the structure of Zheng *et al.* (5AYW[19]) is very similar to that of Gu *et al.* (5D0O[14]) (whole complex Cα RMSD 1.573 Å) only 5D0O[14] was used for comparisons.

Supplementary Table 3. BAM functional assay data. Four replicates of each OmpT experiment from two proteoliposome preparations were used for analysis. T_{50} values were calculated from baselines fitted to the end of the kinetic traces. The mean, standard deviation, and standard error of the mean for each sample are reported below. In addition, a paired Students t-test was performed to compare specific subsets of data, for which p-values are shown.

	T_{50} (sec)						
	Repeat 1	Repeat 2	Repeat 3	Repeat 4	MEAN	ST.DEV	S.E.M
WT	540	500	460	540	510.0	33.17	16.58
WT_{red}	420	440	480	650	497.5	90.66	45.33
WT_{ox}	480	520	470	480	487.5	19.20	9.60
Q-MUT	1590	1420	1050	1440	1375.0	198.81	99.40
Q-MUT_{red}	840	770	840	870	830.0	36.74	18.37
Q-MUT_{ox}	1420	1270	1400	890	1245.0	212.90	106.45
Cys-free	540	460	670	730	600.0	106.07	53.03
Cys-$free_{red}$	370	390	420	580	440.0	82.76	41.38
Cys-$free_{ox}$	460	590	640	700	597.5	88.42	44.21

	T-test
WT vs Q-MUT	0.0030**
WT_{red} vs Q-MUT_{red}	0.0042**
WT_{ox} vs Q-MUT_{ox}	0.0088**
Q-MUT vs Cys-free	0.0141**
WT vs Cys-free	0.2553

Supplementary References

1. Roman-Hernandez, G., Peterson, J.H. & Bernstein, H.D. Reconstitution of bacterial autotransporter assembly using purified components. *Elife* **3**, e04234 (2014).
2. Thoma, J., Bosshart, P., Pfreundschuh, M. & Muller, D.J. Out but not in: the large transmembrane beta-barrel protein FhuA unfolds but cannot refold via beta-hairpins. *Structure* **20**, 2185-90 (2012).
3. McMorran, L.M., Bartlett, A.I., Huysmans, G.H., Radford, S.E. & Brockwell, D.J. Dissecting the effects of periplasmic chaperones on the in vitro folding of the outer membrane protein PagP. *J Mol Biol* **425**, 3178-91 (2013).
4. Burmann, B.M., Wang, C. & Hiller, S. Conformation and dynamics of the periplasmic membrane-protein-chaperone complexes OmpX-Skp and tOmpA-Skp. *Nat Struct Mol Biol* **20**, 1265-72 (2013).
5. Marty, M. et al. Bayesian Deconvolution of Mass and Ion Mobility Spectra: From Binary Interactions to Polydisperse Ensembles. *Anaytical Chemistry* **87**, 44370-4376 (2015).
6. Kremer, J., Mastronarde, D. & McIntosh, J. Computer visualization of three-dimensional image data using IMOD. *Journal of Structural Biology* **116**, 71-76 (1996).
7. Li, X. et al. Electron counting and beam-induced motion correction enable near-atomic-resolution single-particle cryo-EM. *Nature Methods* **10**, 584-590 (2013).
8. Tang, G. et al. EMAN2: an extensible image processing suite for electron microscopy. *Journal of Structural Biology* **157**, 38-46 (2006).
9. Scheres, S. Semi-automated selection of cryo-EM particles in RELION-1.3. *Journal of Structural Viology* **189**, 114-122 (2015).
10. Scheres, S. RELION: implementation of a Bayesian approach to cryo-EM structure determination. *Journal of Structural Biology* **180**, 519-530 (2012).
11. Scheres, S. Beam-induced motion correction for sub-megadalton cryo-EM particles. *Elife* **13**, e03665 (2014).
12. Kucukelbir, A., Sigworth, F.J. & Tagare, H.D. Quantifying the local resolution of cryo-EM density maps. *Nature Methods* **11**, 63-65 (2014).
13. Pettersen, E. et al. UCSF Chimera--a visualization system for exploratory research and analysis. *Journal of Computational Chemistry* **25**, 1605-1612 (2004).
14. Gu, Y. et al. Structural basis of outer membrane protein insertion by the BAM complex. *Nature* **531**, 64-9 (2016).
15. Noinaj, N., Fairman, J.W. & Buchanan, S.K. The crystal structure of BamB suggests interactions with BamA and its role within the BAM complex. *J Mol Biol* **407**, 248-60 (2011).
16. Albrecht, R. & Zeth, K. Structural basis of outer membrane protein biogenesis in bacteria. *J Biol Chem* **286**, 27792-803 (2011).
17. Trabuco, L., Villa, E., Mitra, K., Frank, J. & Schulten, K. Flexible fitting of atomic structures into electron microscopy maps using molecular dynamics. *Structure* **16**, 673-683 (2008).
18. DiMaio, F. et al. Atomic accuracy models from 4.5 Å cryo-electron microscopy data with density-guided iterative local rebuilding and refinement. *Nature Methods* **361**, 361-365 (2015).
19. Han, L. et al. Structure of the BAM complex and its implications for biogenesis of outer-membrane proteins. *Nat Struct Mol Biol* **23**, 192-6 (2016).
20. Bakelar, J., Buchanan, S.K. & Noinaj, N. The structure of the beta-barrel assembly machinery complex. *Science* **351**, 180-6 (2016).
